# Mitogenomic Insight into the Population Genetic Diversity and Phylogeography of Soybean Stink Bug (*Riptortus pedestris*) in China

**DOI:** 10.3390/insects17030337

**Published:** 2026-03-19

**Authors:** Yuxin Zhou, Shusen Shi, Lei Chen, Zhengxiao Du, Yuan Chen, Junkui Ma, Wenbin Wang, Lulu Wang, Yinyue Zhao, Shiyu Zhu, Yu Gao

**Affiliations:** 1 College of Plant Protection, Jilin Agricultural University, Changchun 130118, China; zhouyx@jlau.edu.cn (Y.Z.); sss-63@263.net (S.S.);; 2 Key Laboratory of Soybean Disease and Pest Control, Ministry of Agriculture and Rural Affairs, Changchun 130118, China; 3 Guangxi Academy of Agricultural Sciences, Nanning 530007, China; 4 The Industrial Crop Institute, Shanxi Agricultural University, Fenyang 032200, China; 5 Crop Research Institute, Liaoning Academy of Agricultural Sciences, Shenyang 110161, China; 6 Suzhou Academy of Agricultural Sciences, Suzhou 234000, China; 7 Institute of Food Crops, Yunnan Academy of Agricultural Sciences, Kunming 650205, China

**Keywords:** *Riptortus pedestris*, phylogeography, genetic differentiation, population expansion

## Abstract

*Riptortus pedestris* (Hemiptera, Alydidae) is widely distributed across East Asia, where significant genetic differentiation may occur among geographic populations. To understand the genetic structure, historical dynamics, and formation of geographic distribution patterns in China, we conducted a phylogeographic analysis using three mitochondrial genes (COI, COII, Cytb) from 35 populations. After PCR amplification, we performed genetic diversity analysis, Fst/Nm estimation, phylogenetic reconstruction (ML, BI, NJ), haplotype network, AMOVA, neutrality tests, mismatch distribution, and molecular dating. Results revealed high genetic diversity (Hd > 0.81, π > 0.011), an AT-rich base composition, and faster evolution at the first codon position. Genetic and geographic distances were significantly correlated, with high Fst values indicating strong differentiation, especially between southwestern/southern and other populations. Two main clades were identified: Clade 1 (mainly southern and southwestern China) and Clade 2 (central, northern, northwestern, and northeastern China). A star-like haplotype network and neutrality tests suggested a rapid expansion around 0.019–0.022 Ma (Last Glacial Maximum), and molecular dating estimated the main split at ~0.029 Ma. AMOVA and Gst/Nst confirmed significant phylogeographic structure with most variation among populations. This study provides initial evidence for the genetic differentiation and evolutionary history of *R. pedestris* in China, demonstrating that its population structure was shaped by climatic changes and geographical isolation, providing key insights into its adaptive evolution and dispersal.

## Simple Summary

*Riptortus pedestris* is a serious soybean pest in China. To understand how it spreads and adapts, we analyzed genetic samples from 35 locations nationwide. We found the highest genetic diversity in southern and eastern regions—especially in Guangxi, Guizhou, Yunnan, Jiangxi, Fujian, and Anhui—suggesting these are long-established habitats. In contrast, some northern and isolated populations showed little or no variation, likely due to limited movement and mating between distant groups. Geographic distance strongly influences genetic differences: The farther apart populations are, the less they exchange genes. These results help pinpoint where the pest is most adaptable and likely to evolve resistance to control measures. This knowledge will aid in predicting its spread, improving monitoring, and guiding sustainable pest management to protect soybean crops and support food security in China.

## 1. Introduction

*Riptortus pedestris* Fabricius (Hemiptera: Alydidae) is a polyphagous pest widely distributed across East and Southeast Asia. In recent years, its damage to soybean crops in major production regions of China has intensified, and it has emerged as one of the key threats to sustainable soybean production [1,2]. Both adults and nymphs feed by piercing and sucking sap from stems, leaves, flowers, and pods of leguminous plants, leading to stunted growth, abscission of flowers and pods, shriveled seeds, and even complete yield loss. Severe infestations can induce the ‘green stem syndrome’ in soybeans, significantly reducing both yield and quality [3,4]. Moreover, *R. pedestris* acts as a vector for pathogenic yeasts such as *Eremothecium coryli* and *Eremothecium ashbyi*, which cause seed mold and staining, further diminishing the market value of agricultural products [5,6,7,8].

With ongoing climate change and shifts in agricultural practices, the distribution range of *R. pedestris* has been expanding. Once primarily confined to southern China, it has gradually spread into major soybean-growing regions in the Huang-Huai-Hai, Northeast, and Northwest, transitioning from a secondary to a primary pest and posing a growing threat to China’s soybean industry [2,9]. Predictive modeling using Maxent suggests that under future climate scenarios, the suitable habitat for *R. pedestris* may expand significantly, with new potential distribution areas projected in northeastern China and western India, thereby increasing its risk of outbreak [2]. Although the biology, ecology, and damage characteristics of *R. pedestris* have been relatively well documented [2,10,11,12,13], studies on its population genetic structure, geographic differentiation, and evolutionary history remain limited.

Population genetic diversity serves as the foundation for species adaptation to environmental changes, range expansion, and outbreak dynamics. Higher genetic diversity generally enhances a population’s adaptability to diverse environments, facilitating successful colonization and establishment in new habitats [14,15]. Mitochondrial markers such as COI and Cytb have been widely employed across Hemiptera to assess genetic diversity, delineate phylogeographic structure, and infer evolutionary histories in stink bugs and other true bugs [16,17,18]. Previous studies using mitochondrial COI and Cytb gene fragments have preliminarily elucidated the phylogenetic relationship between *R. pedestris* and its congener *R. linearis*, revealing notable phylogeographic structuring. Populations from Fujian and Zhejiang exhibit relatively high genetic diversity, suggesting these regions may have served as glacial refugia [19]. Park et al. analyzed COI sequences from 42 geographic populations in South Korea and also detected moderate genetic variation and geographic differentiation [20]. However, these studies were constrained by limited sampling coverage, a small number of molecular markers, and insufficient analytical depth, and thus fail to provide a comprehensive understanding of the species’ nationwide genetic architecture and evolutionary trajectory.

Phylogeography, which integrates principles from phylogenetics, population genetics, and biogeography, has become a powerful approach for unraveling the demographic history, dispersal routes, refugial locations, and divergence times of species, particularly in elucidating the mechanisms behind insect distribution patterns [21,22,23]. Mitochondrial genes are widely used in insect phylogeographic studies due to their maternal inheritance, moderate mutation rates, and ease of amplification. Among these, the COI, COII, and Cytb gene regions have proven particularly effective in resolving population genetic structure and historical dynamics [24,25,26,27,28].

Therefore, in this study, we focus on *R. pedestris* and employ sequences from three mitochondrial genes—COI, COII, and Cytb—to systematically analyze genetic diversity, population structure, and phylogenetic lineages across different geographic populations. By integrating molecular clock analyses, we estimate divergence times and historical expansion events, aiming to uncover the origins, evolutionary pathways, and mechanisms underlying the current geographic distribution of this pest. Our findings will enhance understanding of its adaptive evolution and dispersal patterns, provide a scientific basis for regional monitoring, early warning systems, and green control strategies, and contribute valuable insights to the phylogeographic study of coreoid bugs.

## 2. Materials and Methods

### 2.1. Insect Collection and Sample Preparation

**Figure 1:**
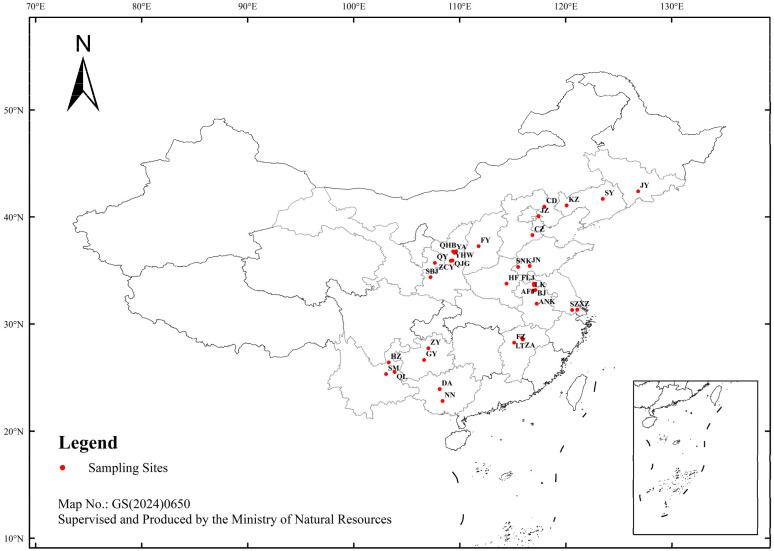
Geographical distribution of *R. pedestris* in China. This map shows the locations of 35 geographic populations from which *R. pedestris* specimens were collected between August 2017 and August 2022; red circles indicate sample collection sites. The map is based on the WGS84 geographic coordinate system and uses a cylindrical equal-area projection. The base map carries the official Chinese map review number GS(2010)1823, approved and supervised by the Ministry of Natural Resources of the People’s Republic of China. Map orientation follows standard geographic convention: north at the top, south at the bottom, west to the left, and east to the right.

Adults and nymphs of *R. pedestris* were collected from soybean fields across 16 provinces (autonomous regions) in China during the peak occurrence periods from 2018 to 2022. A total of 35 geographic populations were sampled (Table S1, Figure 1). Sampling localities and geographic coordinates (longitude E, latitude N) are as follows: Du’an (DA: 108.105° E, 23.933° N) and Nanning (NN: 108.366° E, 22.818° N), Guangxi; Guiyang (GY: 106.628° E, 26.647° N) and Zunyi (ZY: 107.034° E, 27.730° N), Guizhou; Qilin (QL: 103.854° E, 25.524° N), Songming (SM: 103.043° E, 25.327° N), and Huize (HZ: 103.297° E, 26.417° N), Yunnan; Liantang (LT: 115.935° E, 28.545° N), Nanchang (NC: 115.858° E, 28.683° N), and Gao’an (ZA: 115.942° E, 28.564° N), Jiangxi; Fuzhou (FZ: 115.118° E, 28.249° N), Fujian; Hefei Academy of Agricultural Sciences (ANK: 117.246° E, 31.888° N), Baoji (BJ: 117.124° E, 33.164° N), Yongqiao Fengjia (AFJ: 116.987° E, 33.626° N), Fuli Ji (FLJ: 116.977° E, 33.755° N), and Longkang Farm, Bengbu (LK: 116.872° E, 33.101° N), Anhui; Huangfan Farm, Zhoukou (HF: 114.403° E, 33.762° N), Henan; Suzhou (SZ: 120.585° E, 31.300° N) and Xuzhou (XZ: 121.070° E, 31.331° N), Jiangsu; Baoji (SBJ: 107.238° E, 34.363°N), Zhangcunyi, Fuxian County (ZCY: 109.142° E, 35.898° N), Qiaojiagou, Fuxian County (QJG: 109.308° E, 35.924° N), Yanhewan, Ansai (YHW: 109.365° E, 36.766° N), Qinghuabian, Baota District (QHB: 109.654° E, 36.790° N), and Yan’an (YA: 109.495° E, 36.650° N), Shaanxi; Qingyang (QY: 107.643° E, 35.709° N), Gansu; Fenyang (FY: 111.771° E, 37.261° N), Shanxi; Jinan Academy of Agricultural Sciences (SNK: 115.497° E, 35.321° N) and Jining (JN: 116.587° E, 35.415° N), Shandong; Cangzhou (CZ: 116.839° E, 38.305° N) and Chengde (CD: 117.963° E, 40.953° N), Hebei; Jizhou (JZ: 117.408° E, 40.047° N), Tianjin; Kazuo (KZ: 120.068° E, 41.074° N) and Shenyang (SY: 123.473° E, 41.683° N), Liaoning; and Jingyu (JY: 126.813° E, 42.389° N), Jilin. Collected individuals were used to establish laboratory-reared colonies for subsequent experiments. Rearing conditions [29] were maintained at a photoperiod of 16 h:8 h (L:D), temperature of 25 ± 1 °C, and relative humidity of 60–70%. Soybean cultivar ‘Jinong 38’ was used as the host plant in potted cultures.

### 2.2. Genomic DNA Extraction

Genomic DNA was extracted using the Ezup Column Animal Genomic DNA Kit (Sangon Biotech, Shanghai, China), following the manufacturer’s protocol. For each geographic population, five adult males and five adult females were selected for DNA extraction. The concentration and purity of extracted DNA were determined using a NanoDrop spectrophotometer (A260/A280 ratio, Thermo Fisher Scientific, Waltham, MA, USA) to ensure suitability for downstream PCR amplification. Additionally, a small aliquot of each DNA sample was subjected to 1% agarose gel electrophoresis (run on a DYY-7C electrophoresis apparatus, Beijing Liuyi Instrument Factory, Beijing, China) and visualized using a Gel Imaging System (Bio-Rad, Hercules, CA, USA) to assess DNA integrity and detect degradation. High-quality DNA samples were stored at −20 °C (Midea BCD-196SQMK refrigerator, Midea, Beijiaozhen, China) for subsequent PCR amplification.

### 2.3. PCR Amplification and Sequencing

Mitochondrial gene fragments—COI, COII, and Cytb—were amplified via PCR. Genomic DNA was extracted from whole individual insects using a standard protocol, ensuring sufficient template quality for amplification. Primers were designed based on previously published sequences [19,21] (Table S2). The 50 µL PCR reaction mixture contained: 25 µL ddH_2_O, 18 µL Taq-PCR Mix (Sangon Biotech, Shanghai, China), 2 µL each of forward and reverse primers, 2 µL DNA template, and 1 µL MgCl_2_. Amplification was performed on a MG48+ Thermal Cycler (Hangzhou, China) under conditions specified by the manufacturer. PCR products were visualized on 1% agarose gels. Amplicons with clear bands and correct sizes were sent to BGI (BGI Genomics Co., Ltd., Shenzhen, China) for bidirectional Sanger sequencing.

### 2.4. Sequence Processing and Alignment

Raw sequencing data from BGI were quality-checked and forward/reverse reads were manually assembled and corrected using Seqman v12.3 [30]. Assembled sequences were saved in .txt format. Sequence identity and reliability were confirmed via BLAST analysis against the GenBank database (https://blast.ncbi.nlm.nih.gov/, accessed 5 January 2026). Sequences were converted to .fas format using MEGA 7.0 [31]. Multiple sequence alignment was performed using the MAFFT (v7.526) algorithm [32] implemented in PhyloSuite v1.2.2 under the ‘Normal’ mode with the G-INS-i strategy. Aligned sequences were further trimmed and adjusted in MEGA 7.0 [31] and saved in .fas format for downstream analyses.

### 2.5. Genetic Diversity and Population Structure Analysis

Genetic diversity parameters were calculated using DnaSP v5.0 [33], including number of haplotypes (Nh), haplotype diversity (Hd), nucleotide diversity (π), sequence diversity (K), and number of polymorphic sites (S). Population pairwise genetic distances (p-distances) were computed in MEGA 7.0 [31] with 1000 bootstrap replicates. Genetic differentiation among populations (Fst) and gene flow (Nm) were estimated using Arlequin v3.5 [34], where Nm = (1 − Fst)/(4 × Fst). Fst values were interpreted as follows [35]: Fst < 0.05 indicates low differentiation, 0.05 ≤ Fst < 0.15 indicates moderate differentiation, and Fst ≥ 0.25 indicates high differentiation. An Nm value > 1 suggests gene flow, while Nm > 4 indicates panmixia.

### 2.6. Phylogenetic and Haplotype Network Analyses

*Halyomorpha halys* and *Lygus pratensis* were designated as outgroups. The best-fit substitution model for each gene and the concatenated dataset was selected using ModelFinder [36] in PhyloSuite v1.2.2 [37]. Maximum Likelihood (ML) trees were constructed using IQ-TREE [38], and Bayesian Inference (BI) trees were generated using MrBayes 3.2 [39] for COI, Cytb, and the concatenated sequence. For COII, a Neighbor-Joining (NJ) tree was constructed in MEGA 7.0 [31]. The concatenated dataset was generated by aligning each gene separately with MAFFT [32], trimming and aligning in MEGA 7.0 [31], concatenating using PhyloSuite’s Concatenate function, and partitioning with PartitionFinder [37] to assign optimal models to each gene. Final ML and BI trees were built using IQ-TREE [38] and MrBayes 3.2 [39], respectively. Haplotype networks for COI, COII, Cytb, and the concatenated dataset were constructed using the median-joining algorithm in POPART v1.7. This method is particularly suitable for visualizing relationships among closely related haplotypes and inferring potential ancestral nodes within intraspecific datasets. Both ML and NJ phylogenetic trees were assessed with 1000 bootstrap replicates.

### 2.7. Population Historical Dynamics and Divergence Time Estimation

Analysis of Molecular Variance (AMOVA) was performed in Arlequin v3.5 [34] to assess population genetic structure. The presence of phylogeographic structure was evaluated by comparing Gst and Nst values in DnaSP v5.0 [33]; a significantly higher Nst than Gst indicates phylogeographic signal. Population demographic history was inferred through neutrality tests (Tajima’s D [40] and Fu’s Fs [41]) and mismatch distribution analysis, both conducted in Arlequin v3.5 [42] with 1000 permutations under a sudden expansion model. Significance of the Sum of Squared Deviations (SSD) and Harpending’s raggedness index (r) was used to assess population expansion. The time since population expansion (t) was estimated using the formula τ = 2 ut, where τ was derived from mismatch analysis in Arlequin, u is the mutation rate (u = mTμ), mT is the sequence length (2158 bp), μ is the substitution rate (0.023 per million years) [43], and t is the number of generations per year (3).

Divergence times were estimated using BEAST v1.10.4 [42]. The concatenated sequence in .nex format was imported into BEAUti v1.10.4. The outgroup was specified, the substitution model was set to GTR + G, and a strict molecular clock was applied with a rate of 0.023 substitutions per site per million years [44]. The MCMC chain length was set appropriately with sampling every 1000 steps. The resulting XML file was run in BEAST v1.10.4 [42]. Effective Sample Size (ESS) values were checked using Tracer v1.7 [45] (all > 200). After discarding 10% as burn-in, a maximum clade credibility (MCC) tree was generated using TreeAnnotator v1.10.4 and visualized in FigTree v1.1.1 [46].

## 3. Results

### 3.1. Base Composition and Genetic Diversity of Concatenated Mitochondrial Gene Sequences

The concatenated 2158 bp sequence was assembled from 350 individuals (10 from each of 35 populations), all of which provided complete sequences for COI, COII, and Cytb. Base composition analysis (Table 1) revealed a pronounced A + T bias: T = 38.67%, A = 32.73%, G = 13.68%, C = 14.92%, resulting in A + T = 71.64% and G + C = 28.40%. Among codon positions, the first had the highest A + T content (77.70%), followed by the third (61.20%), and the second (45.13%), indicating the third position contributes most to A + T bias. For individual gene-level base composition, see Tables S3, S6 and S9 (Supplementary Materials, Sections S3.1.1, S3.2.1 and S3.3.1).

Substitution analysis (Table 2) showed 27 transitions (si) and 3 transversions (sv), yielding a transition/transversion ratio (R) of 9. No transversions were detected at the second or third codon positions, confirming their high evolutionary conservation. Among 16 dinucleotide combinations, TT and AA were most abundant, followed by GG and CC (~1/3 abundance), while heterologous pairs were rare. Detailed substitution frequency statistics for each gene (COI, COII, and Cytb) are provided in Tables S4, S7 and S10 (Supplementary Materials, Sections S3.1.1, S3.2.1 and S3.3.1).

Genetic diversity analysis using DnaSP v5.0 (Table 3) revealed extremely high overall diversity: a total of 184 haplotypes (Hap) were identified in the concatenated dataset, *Hd* = 0.9818, *π* = 0.01378, *K* = 29.74133, and *S* = 194 polymorphic sites. Haplotype diversity was zero in Zunyi (ZY), but reached 1.000 in Gao’an (GA, Jiangxi) and Kazuo (KZ, Liaoning). Populations with *K* > 10 included Nanning (NN, Guangxi; *K* = 12.47), Guiyang (GY, Guizhou; *K* = 24.38), Qilin (QL, Yunnan; *K* = 14.20), Songming (SM, Yunnan; *K* = 12.16), Liantang (LT, Jiangxi; *K* = 30.98), Nanchang (NC, Jiangxi; *K* = 39.31), Gao’an (GA, Jiangxi; *K* = 20.40), Fuzhou (FZ, Fujian; *K* = 40.36), Hefei (HF, Anhui; *K* = 40.42), Fengjia (AFJ, Anhui; *K* = 15.49), and Bengbu (BB, Anhui; *K* = 16.64). Fuzhou (FZ) and Hefei (HF) showed *K* values approaching 40 and the highest *π* (0.0187). High-diversity populations were concentrated in Southwest and East China. Genetic diversity metrics for each individual gene (COI, COII, and Cytb) are provided separately in Tables S5, S8 and S11 (Supplementary Materials, Sections S3.1.2, S3.2.2 and S3.3.2).

### 3.2. Inter-Population Genetic Distances, Gene Flow, and Genetic Differentiation

**Figure 2:**
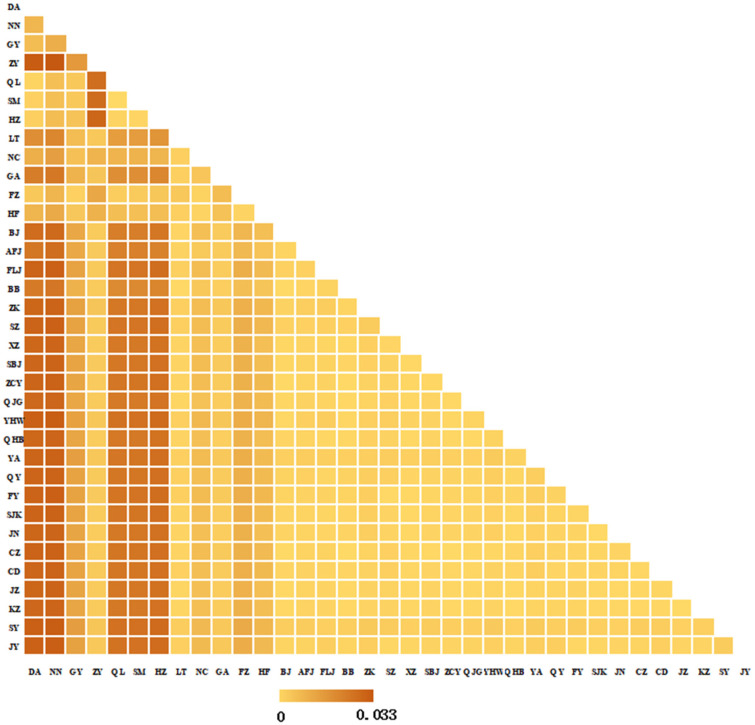
P distances among 35 populations. The heatmap displays the genetic divergence between each pair of populations, with darker orange shades indicating higher genetic distances (up to 0.033), and lighter yellow shades indicating lower distances. Diagonal cells represent self-comparisons (distance = 0).

Pairwise genetic distances (p-distances) calculated in MEGA 7.0 ranged from 0.000 to 0.033 (Figure 2). Populations from Southwest and South China showed relatively large distances (0.003–0.032) from those in Central, East, North, Northwest, and Northeast China. Zunyi (ZY) was particularly distant from Southwest/South populations (0.016–0.033), with the largest distances to Du’an (DA, 0.032) and Nanning (NN, 0.033)—the highest among all pairwise comparisons. In contrast, most populations in Central, East, North, Northwest, and Northeast China showed small distances (0.000–0.012), indicating high genetic similarity. For gene-specific pairwise p-distances based on COI, COII, and Cytb individually, see Figures S1, S7 and S13 (Supplementary Materials, Sections S3.1.3, S3.2.3 and S3.3.3).

**Figure 3:**
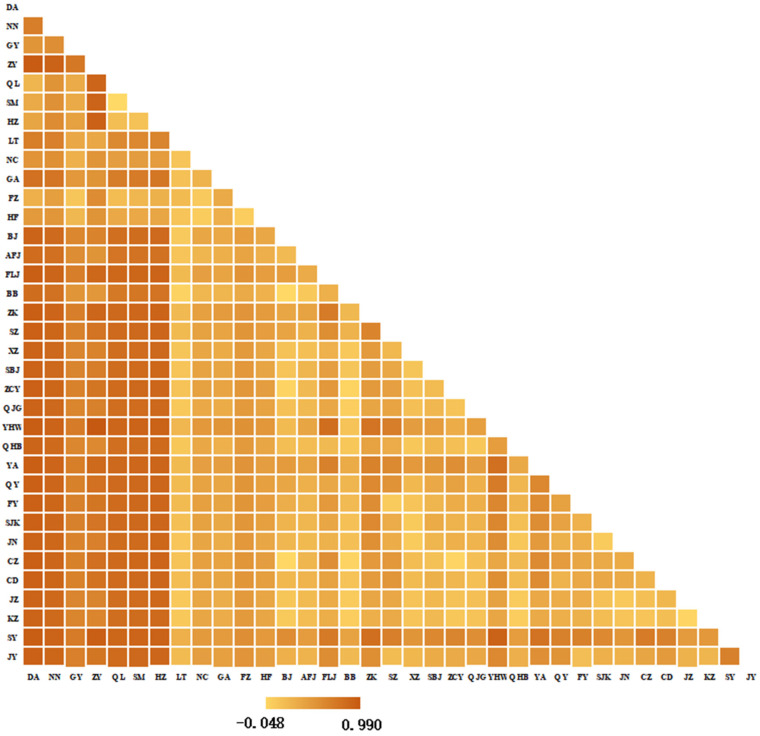
*Fst* values among 35 populations of the COI, COII, and Cytb genes. The heatmap displays genetic differentiation between populations, with darker orange shades indicating higher *Fst* values (up to 0.990), reflecting greater genetic divergence, and lighter yellow shades indicating lower *Fst* values (down to −0.048), suggesting potential gene flow or sampling bias. Diagonal cells represent self-comparisons and are set to zero.

**Figure 4:**
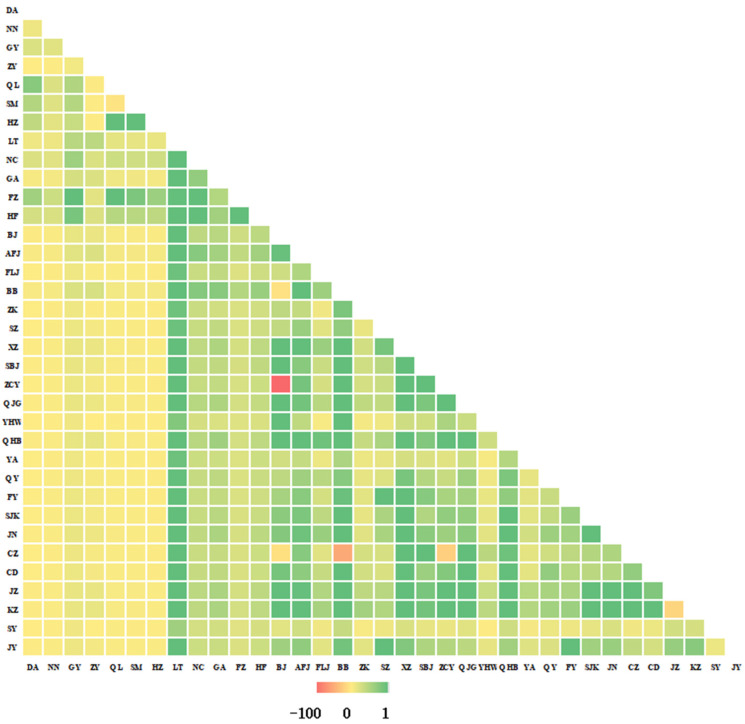
Nm values among 35 populations of the COI, COII, and Cytb genes. The heatmap displays the number of migrants per generation between each pair of populations, with darker green shades indicating higher gene flow (up to 4.526), lighter yellow shades indicating lower gene flow, and red cells representing negative values. Diagonal cells are set to zero.

*Fst* and *Nm* values were calculated using Arlequin v3.5. *Fst* ranged from −0.048 to 0.990 (Figure 3). High *Fst* values (0.287–0.990) were observed between Southwest/South China and other regions, indicating strong differentiation. Zunyi (ZY) showed particularly high *Fst* (0.743–0.967) with Southwest/South populations, suggesting near-complete reproductive isolation. Negative *Fst* between Qilin (QL) and Songming (SM, Yunnan) implied frequent gene flow. Gene-wise Fst matrices for COI, COII, and Cytb are provided in Supplementary Figures S2, S8 and S14 (Supplementary Materials, Sections S3.1.4, S3.2.4 and S3.3.4). *Nm* values ranged from −107.088 to 4.526 (Figure 4). Most *Nm* > 1 in East, North, Northwest, and Northeast China, indicating gene flow; values > 4 suggest panmixia. Corresponding Nm estimates for each individual gene (COI, COII, Cytb) are shown in Supplementary Figures S3, S9 and S15 (Supplementary Materials, Sections S3.1.4, S3.2.4 and S3.3.4).

### 3.3. Phylogenetic and Haplotype Network Analyses of Concatenated Sequences

Before tree construction, Xia’s test for substitution saturation (DAMBE) showed ISS values significantly lower than ISS.C (*p* < 0.001), regardless of third-codon inclusion, indicating no saturation and suitability for phylogenetic inference.

**Figure 5:**
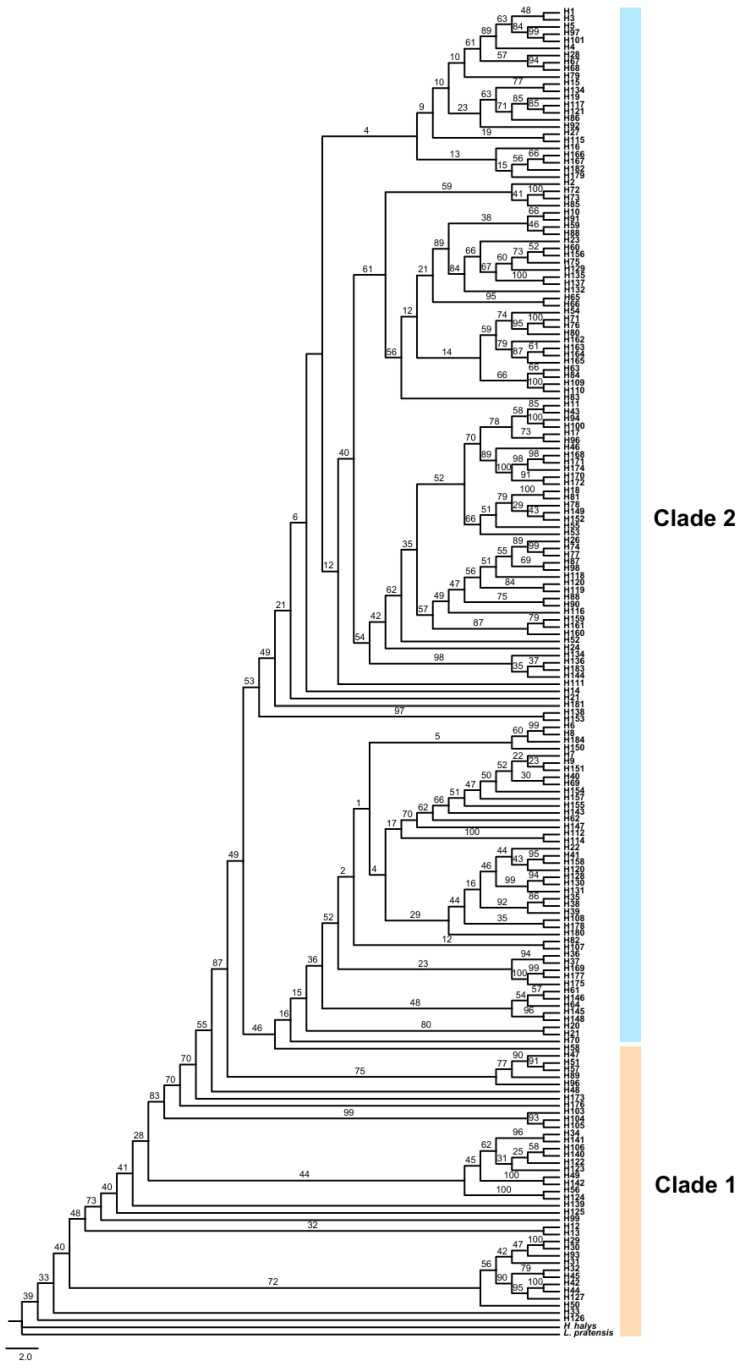
ML phylogenetic trees of the COI, COII, and Cytb genes based on 35 populations of *R. pedestris*. Terminal tips represent haplotypes (H1–H184); bootstrap support values (1000 replicates) ≥70% are shown at nodes. The tree is rooted with L. *protensisis* and *H. halys* and divided into Clade 1 (orange) and Clade 2 (blue). Scale bar = 0.02 substitutions per site.

**Figure 6:**
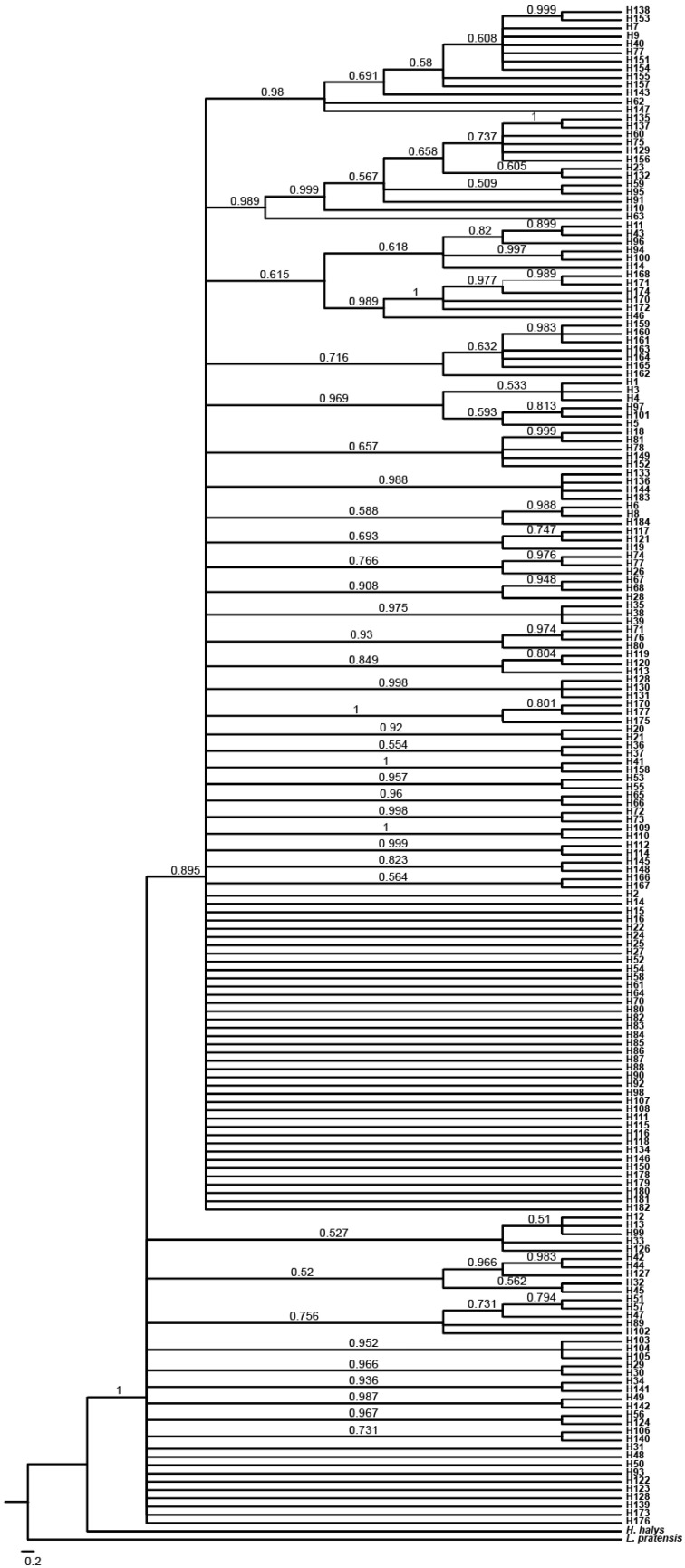
ML phylogenetic trees of the COI, COII, and Cytb genes based on 35 populations of *R. pedestris*. Terminal tips represent haplotypes (H1–H184); posterior probabilities ≥0.95 are shown at nodes. The tree is rooted with *H. halys* and *L. pratensis*. Scale bar indicates nucleotide substitutions per site.

ML and BI trees based on concatenated sequences showed highly consistent topologies (Figure 5 and Figure 6), dividing *R. pedestris* into two main clades. Clade 1 (38 haplotypes) included all samples from Guangxi (DA, NN), Guizhou (GY), and Yunnan (QL, SM, HZ), plus subsets from Jiangxi (LT, NC, GA), Fujian (FZ), and Anhui (HF, BJ, BB, AFJ). Clade 2 (146 haplotypes) encompassed all populations from Central, North, Northwest, and Northeast China, as well as partial samples from East and Southwest (ZY). This clade showed complex internal structure with no clear geographic subdivision. The unique haplotype H184 (ZY) formed a distinct cluster.

**Figure 7:**
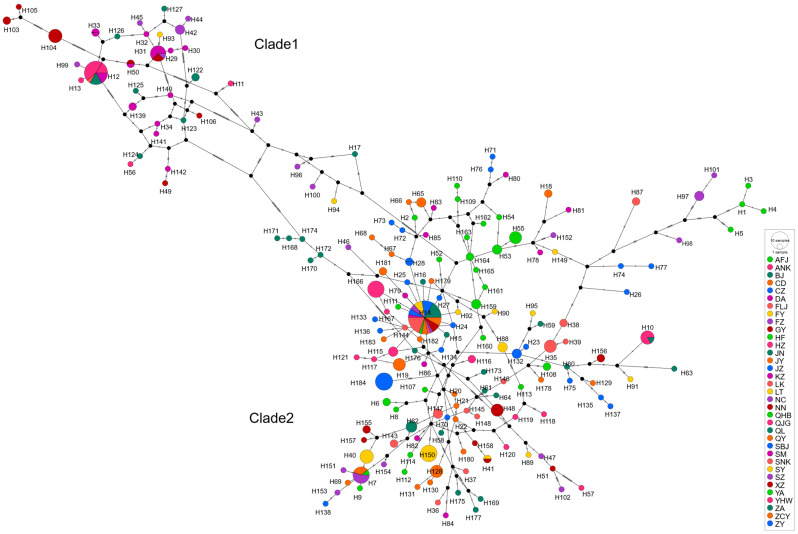
Haplotype network of the COI, COII, and Cytb genes based on 35 populations of *R. pedestris*. Constructed using the TCS algorithm in POPART. Circles represent observed haplotypes, with size proportional to frequency. Each connecting line represents a single nucleotide mutation. Colors denote geographic origin (see legend). The network is divided into two major clades: Clade 1 and Clade 2.

The haplotype network (Figure 7) supported this split, with 68 median vectors. Clade 1 (38 haplotypes, 24 vectors) had multiple central haplotypes (e.g., H11, H17, H94, H96, H100), suggesting a polyphyletic ancestral origin. H103–H105 (NN) were peripheral, indicating genetic distinctiveness. Clade 2 (146 haplotypes, 44 vectors) had central haplotype H14, shared by 13 populations, likely ancestral. Its star-like, nested structure suggests rapid recent expansion. Haplotype H184 (ZY) was isolated from northern clusters.

Individual gene analyses for COI, COII, and Cytb are provided in the Supplementary Materials, Sections S3.1.4, S3.2.4 and S3.3.4 (Figures S4, S10 and S16: ML Phylogenetic trees; Figures S5, S11 and S17: BI Phylogenetic trees; Figures S6, S12 and S18: Haplotype networks based on 35 populations of *R. pedestris*), which show largely concordant patterns with the concatenated sequence analysis presented in the main figures.

### 3.4. Population Genetic Structure, Demographic History, and Divergence Time Estimation

Given the extremely high concordance among the three mitochondrial genes (COI, COII, Cytb) and their concatenated sequence (pairwise *r* ≥ 0.95; see Supplementary Table S12), all subsequent analyses were based on the concatenated dataset to maximize phylogenetic resolution and avoid redundancy. The 35 populations were grouped into three regions: Group 1 (South China: DA, NN), Group 2 (Southwest China: GY, ZY, QL, SM, HZ), and Group 3 (Broadly Northern: all others). AMOVA (Table 4) revealed 58.77% of genetic variation occurred among groups, 18.24% among populations within groups, and 22.99% within populations. Total *Fst* = 0.77014 (*p* < 0.001), indicating significant intergroup differentiation.

Phylogeographic structure was tested by comparing *Gst* and *Nst*: mean *Nst* (0.629) was significantly greater than *Gst* (0.220). Pairwise comparisons (e.g., Group 3 vs. Group 1: *Nst* = 0.71088, *Gst* = 0.01951) confirmed a strong association between genetic and geographic structure (Table 5).

**Figure 8:**
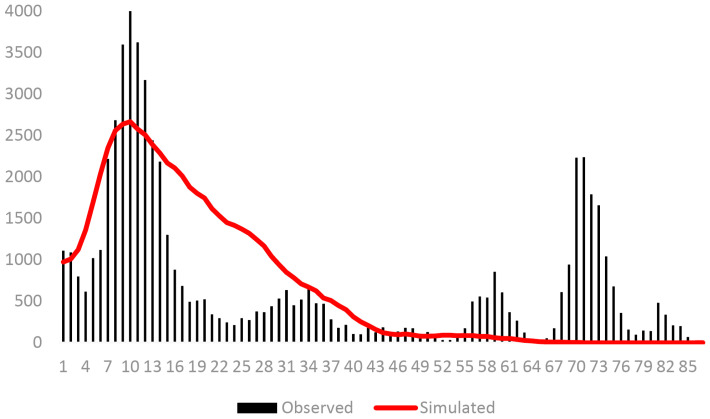
Mismatch distribution analysis diagram of 35 populations of *R. pedestris*.

**Figure 9:**
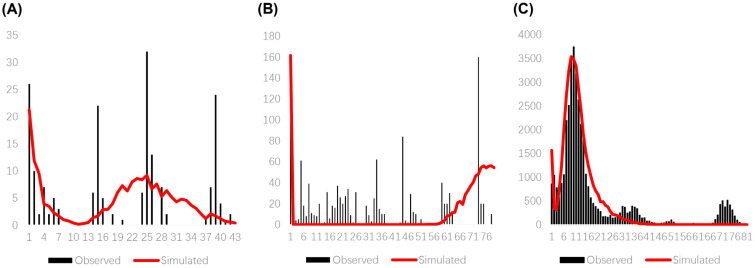
Mismatch distribution analysis diagram of Group 1 (**A**), Group 2 (**B**) and Group 3 (**C**) of 35 populations of *R. pedestris*.

Neutrality tests and mismatch distribution analyses (Table 6, Figure 8 and Figure 9) revealed demographic history. Overall, Fu’s *Fs* was significantly negative (−23.4844), and mismatch distribution was unimodal, indicating a historical population expansion ~0.019 Ma (Late Pleistocene, LGM). Groups 1 and 2 showed positive Tajima’s *D* and Fu’s *Fs* and a multimodal mismatch, suggesting stability. Group 3 had a significantly negative Fu’s *Fs* (−23.64885) and a unimodal mismatch, indicating a recent expansion ~0.022 Ma (LGM).

**Figure 10:**
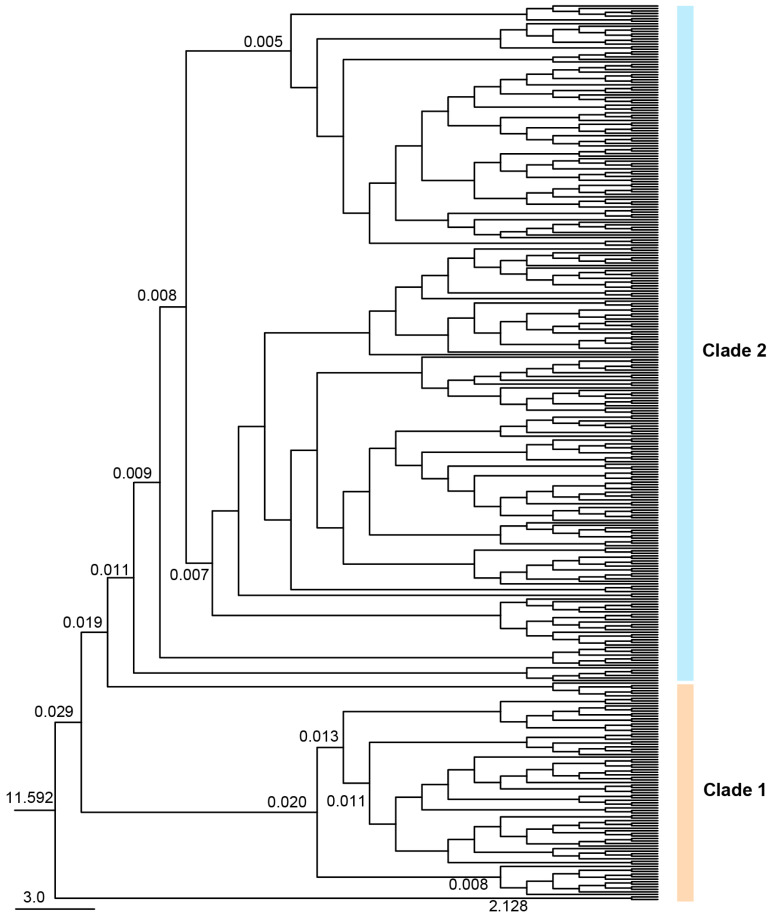
The populations’ divergence times of *R. pedestris*. Terminal tips represent haplotypes; node labels indicate estimated divergence times in millions of years ago (Mya). The tree is divided into two major clades: Clade 1 (orange) and Clade 2 (blue). Scale bar indicates time in Mya.

Divergence times were estimated using BEAST (Figure 10). *R. pedestris* diverged from the outgroup ~11.529 Ma. Clade 1 and Clade 2 split ~0.029 Ma (95% CI: 1.732–130.707 kya), corresponding to the late MIS3 (Late Pleistocene). Subsequent divergences occurred in the Late Pleistocene: ~0.020 Ma within Clade 1, ~0.011 Ma for Nanning (NN), and ~0.019 Ma within Clade 2. These results suggest Quaternary climatic fluctuations were key drivers of population differentiation and expansion in *R. pedestris*.

## 4. Discussion

This study investigated the base composition of mitochondrial genes and their concatenated sequences from 35 geographic populations of *R. pedestris*. The results showed that the A + T content was significantly higher than the G + C content, exhibiting a pronounced AT bias, which is consistent with the base composition characteristics of insect mitochondrial gene sequences [47]. The overall number of transitions between bases was greater than the number of transversions. Among the first, second, and third codon positions, the numbers of transitions and transversions at the second and third positions were lower than at the first position, indicating that the bases at the second and third codon positions are relatively conserved during evolution, while the first position evolves faster. This result aligns with the characteristic of relatively rapid evolutionary rates in insect mitochondrial genes [48].

Analysis of haplotypes using DnaSP v.5.0 [33] software revealed that, based on the COI, COII, Cytb, and concatenated sequences of 35 geographic populations of *R. pedestris*, 56, 20, 74, and 184 haplotypes were obtained, respectively. The overall number of haplotypes is relatively high, consistent with the characteristic that insects generally have more haplotypes than other animals [49]. The COII gene had relatively fewer haplotypes, possibly because the COII gene fragment in *R. pedestris* is inherently shorter and contains fewer mutable base sites. The haplotype diversity (Hd > 0.5) and nucleotide diversity (π > 0.005) of the three mitochondrial genes and the concatenated sequence were all high, indicating a long evolutionary history and stable population, large population size, wide distribution range, and rich genetic diversity, which suggests high differentiation potential and strong adaptability to the environment [50]. However, there was significant variation in nucleotide diversity among different populations. Populations located around 30° N latitude exhibited higher nucleotide diversity, specifically those from Nanning, Guangxi (NN); Guiyang, Guizhou (GY); Qilin, Yunnan (QL); Songming, Yunnan (SM); Liantang, Jiangxi (LT); Nanchang, Jiangxi (NC); Gao’an, Jiangxi (GA); Fuzhou, Fujian (FZ); Hefei, Anhui (HF); Fengjia, Yongqiao District, Anhui (AFJ); and Bengbu, Anhui (BB). This indicates that these populations have a longer history and have accumulated more genetic variation. This result is similar to Lü Minhua’s study on the genetic diversity of *R. pedestris*, which also found high nucleotide diversity in populations from Fujian and Zhejiang [19]. The differences between the studies might be due to this study having a broader and more concentrated sampling distribution, allowing the two studies to complement each other and enrich the research findings on *R. pedestris*. In this study, not all populations exhibited high haplotype and nucleotide diversity; for the COI gene, the populations from Zunyi, Guizhou (ZY), Zhoukou, Henan (ZK), and Shenyang, Liaoning (SY) had zero haplotype and nucleotide diversity. For the COII gene, the populations from Zunyi, Guizhou (ZY), Yanhewan, Ansai, Shaanxi (YHW), and Jining, Shandong (SJN) had zero diversity, and for the Cytb gene, the population from Zunyi, Guizhou (ZY) had zero diversity. This might be because the geographical environments of these locations are relatively unique, blocking gene flow between *R. pedestris* in these areas and populations in other regions. Although our per-population sample size (n = 10) may limit detection of low-frequency haplotypes, it aligns with effective sampling strategies in recent phylogeographic studies of migratory hemipterans [51].

In this study, the results from the three mitochondrial genes and their concatenated sequences were similar. The genetic distance analysis among the 35 geographic populations showed that populations geographically farther apart also had greater genetic distances. For example, the populations from Du’an, Guangxi (DA) and Nanning, Guangxi (NN) in the southwest region had large geographical distances from the populations in the northeast region, Shenyang, Liaoning (SY) and Jingyu, Jilin (JY), with genetic distances reaching as high as 0.037. In contrast, populations in the central, northwest, north, and northeast regions had smaller genetic distances among themselves, all less than 0.007. It is common for geographical distance to cause genetic differences among populations. Typically, gene exchange tends to occur with geographically proximate populations, leading to gene flow. For populations at distant ends, opportunities for gene exchange are fewer, leading to differentiation due to ‘geographical isolation’. The greater the geographical distance, the larger the genetic difference between populations. Gene exchange between adjacent populations makes their genetic structures more similar. This is similar to Bai Yi’s research results [49] and consistent with Lü Minhua’s genetic analysis of *R. pedestris*, which indicated that the widespread *R. pedestris* has relatively large intraspecific genetic distances [19]. The genetic distance between Du’an, Guangxi (DA), Nanning, Guangxi (NN) and Zunyi, Guizhou (ZY) in the southwest region was as high as 0.037, possibly because Zunyi (ZY) is at a relatively low altitude, located on the slope of the Yunnan-Guizhou Plateau, with large terrain undulations and complex landform types. The Dalaoshan Mountains bisect the Zunyi area into southern hills and basins and northern canyons, and this rugged topography may limit gene flow between the ZY population and other southwestern populations, potentially contributing to its high genetic divergence. The average genetic differentiation indices (Fst) for the COI, COII, and Cytb genes and the concatenated sequence of *R. pedestris* were 0.691, 0.476, 0.631, and 0.627, respectively; the gene flow (Nm) values were 0.11, 0.28, 0.15, and 0.15, respectively, indicating that some populations have reached a significant differentiation level, meaning the genetic differentiation between the two clades is relatively obvious, which is also consistent with Lü Minhua’s research results [19]. Overall, the Fst values for populations in the southwest and south regions were higher compared to other geographic populations, specifically the populations from Du’an, Guangxi (DA), Nanning, Guangxi (NN), Guiyang, Guizhou (GY), Zunyi, Guizhou (ZY), Qilin, Yunnan (QL), Songming, Yunnan (SM), and Huize, Yunnan (HZ). This may be related to the limitations of the mountainous environment of the Yunnan-Guizhou Plateau, which increases gene loss and homozygosity in populations, resulting in smaller gene flow and more significant genetic differentiation. However, for the Cytb gene, the Fst values for the populations from Zhoukou, Henan (ZK) and Yanhewan, Ansai, Shaanxi (YHW) were also higher, suggesting that the Qinling Mountains might also have some barrier effect on *R. pedestris*. This result is consistent with Li Yisong’s study on the phylogeography of the oriental fruit moth, which showed that the oriental fruit moth can be divided into two evolutionary clades, separated by the Qinling Mountains and surrounding mountains and rivers [52]. Most *R. pedestris* populations in the central, east, north, northwest, and northeast regions have smaller Fst values and larger Nm values, indicating that gene flow exists among these populations, and that for some population pairs, the absolute Nm value is greater than 4, indicating frequent gene exchange.

Studies have shown that a star-like radiating distribution in a haplotype network diagram indicates that the population is growing and developing, and new mutations are likely to arise from the more abundant haplotypes. This research result is consistent with Lü Minhua’s findings [19]. In this study, the haplotype networks of the COI, COII, and Cytb genes and their concatenated sequences were similar, all dividing *R. pedestris* into two clades. The haplotype pattern in Clade 2 was a star-like radiating distribution, primarily including populations from the central, east, north, northwest, and northeast regions, indicating that these populations are in a continuous expansion state. Some researchers believe that shared haplotypes are stable haplotypes that have been selected by the natural environment and are well-adapted, and shared haplotypes between populations represent a common ancestor [53]. In the concatenated sequence of the three mitochondrial genes of *R. pedestris*, there were 9 shared haplotypes, accounting for 4.89% of all haplotypes. The most abundant haplotype was H14, with 36 individuals, from Fuzhou, Fujian (FZ), Baoji, Anhui (BJ), Bengbu, Anhui (BB), Xuzhou, Jiangsu (XZ), Suzhou, Jiangsu (SZ), Qinghuabian, Baota District, Shaanxi (QHB), Zhangcunyi, Fuxian, Shaanxi (ZCY), Qingyang, Gansu (QY), Cangzhou, Hebei (CZ), Fenyang, Shanxi (FY), Jining, Shandong (SJN), Jizhou, Tianjin (JZ), and Kazuo, Liaoning (KZ). Geographically, these populations are mainly distributed in the central, east, north, northwest, and northeast regions, suggesting that these populations may have evolved from the same ancestor.

In the population historical dynamics analysis of *R. pedestris*, the results of neutrality tests and mismatch distribution were consistent. When the 35 geographic populations were analyzed as a whole, the results indicated that the population underwent a rapid expansion in history, with the expansion time estimated at approximately 0.019 Ma, corresponding to the Last Glacial Maximum (LGM). The results for Group 1 and Group 2 showed stable populations with no expansion. The results for Group 3 showed a recent expansion, with the expansion time estimated at approximately 0.022 Ma, also corresponding to the LGM. During this period, global temperatures dropped rapidly, polar and alpine glaciers extended continuously, and the climate shifted from warm and humid to dry and cold. However, due to China’s relatively low latitude, the impact of the glacial period was less severe. Lower altitude regions were relatively more suitable for survival and more likely to serve as glacial refugia; therefore, the impact of the LGM on the expansion of *R. pedestris* was relatively small [54]. The phylogeographic pattern observed in *R. pedestris*—with high southern diversity, post-glacial northward expansion, and limited impact of the Qinling Mountains—is broadly consistent with findings in other widespread Chinese agricultural pests such as *Callosobruchus chinensis* [55] and *Carposina sasakii* [56], suggesting a common response to Pleistocene climate oscillations and topography across taxa.

*R. pedestris* and *R. linearis* belong to the genus *Riptortus*, which are southern-dwelling species. Outside of China, they are distributed in East and Southeast Asia, but *R. linearis* has a distribution further south. Based on the distribution of *R. pedestris* in neighboring countries of China, centered on the Yunnan-Guizhou Plateau, its presence is recorded in Myanmar, India, Sri Lanka, and Pakistan to the west and northwest of the plateau, and in Thailand, Malaysia, and Indonesia to the south. This suggests that *R. pedestris* within China may have originated from these two directions. One branch may have entered Yunnan, China, from Sri Lanka, India, Pakistan, and Myanmar, then diffused eastward and northward. Another branch may have entered Yunnan and Guangxi, China, from Indonesia, Malaysia, and Thailand, then diffused eastward and northward. This is similar to Bai Yi’s speculation on the origin of *Trilophidia annulata* within China [53], but this is only a preliminary inference, and the results require further investigation.

This study estimated the population divergence time of different *R. pedestris* clades based on a mitochondrial gene average substitution rate of 0.023 per million years. The results showed that the initial divergence of *R. pedestris* occurred at 0.029 Ma, corresponding to the transition period from MIS3 to MIS2 of the last glacial period, when the climate shifted from warm and humid to cold and dry, glaciers gradually expanded, and sea levels dropped [57,58]. Lü Minhua estimated the divergence time of the two branches to be between 2.6 and 4.3 Ma [19], and the significant difference in results might be due to different grouping ranges used when estimating population divergence time. In this study, southern and southwestern populations were grouped into one clade, and other geographic populations into another. During the transition from MIS3 to MIS2 of the last glacial period, the climate had a significant impact on the distribution of plants and animals. Studies have shown that the alternating cold and warm periods of the Pleistocene (2.58–0.01 Ma) drove population differentiation in many biological groups [59]. *R. pedestris* was preliminarily divided into two evolutionary clades based on geography, with Clade 1 diverging earlier than Clade 2. In Clade 1, populations from Qilin, Yunnan (QL), Songming, Yunnan (SM), Huize, Yunnan (HZ), Du’an, Guangxi (DA), and Nanning, Guangxi (NN) clustered into one branch, with a divergence time of approximately 0.013 Ma; Nanning, Guangxi (NN) formed a separate branch with a divergence time of approximately 0.011 Ma. It is speculated that the southwestern region might be the origin and glacial refuge of *R. pedestris*, and as the climate gradually warmed, it subsequently dispersed to the central, east, north, northwest, and northeast regions. This is consistent with Chen Keke’s speculation that *Colias fieldi* and *Colias erate* originated in southwestern China [60]. Clade 2 has a larger number of haplotypes and a multi-layer nested structure, with complex relationships among haplotypes, and haplotypes from different geographic populations often cluster together. We speculate that the drastic climatic turmoil of the Pleistocene and geographical isolation led to genetic differentiation among populations, and when conditions were suitable, rapid merging of populations formed hybrid zones [61,62]. Insect geographical regionalization suggests that the Qinling Mountains create geographical isolation between different populations. However, in this study, the barrier effect of the Qinling Mountains on *R. pedestris* populations was not very obvious. The Qinling Mountains geographically separate Shaanxi, Gansu, and Henan populations from other populations, but the haplotypes cannot be completely separated, indicating that there is some gene flow between populations on the northern and southern sides of the Qinling Mountains, which is consistent with Bai Yi’s study on the phylogeographic differentiation of *Trilophidia annulata* based on mitochondrial genes COII and ND5 [49]. In the two evolutionary clades, haplotypes from the same geographic population often cluster together, because *R. pedestris* is distributed in different geographical environments, and due to mountainous limitations, the distribution of suitable hosts, and unique climatic conditions, *R. pedestris* forms small groups. For example, the terrain of Nanning, Guangxi, is typical mountainous, hilly, and basin terrain, which limits the migration and diffusion of the *R. pedestris* population, causing the Nanning (NN) population to cluster as a separate branch within Clade 1. However, because mitochondrial DNA is maternally inherited and represents a single locus, our estimates of gene flow, divergence times (~0.01–0.03 Ma), and demographic expansion may be biased—particularly if male-mediated dispersal, selection on mtDNA, or nuclear-mitochondrial discordance are present, all of which are common in insects.

Although this study, based on mitochondrial genes (COI, COII, and Cytb), reveals a pronounced phylogeographic structure in *R*. *pedestris*, this approach is widely used in insect population genetics and offers several advantages—such as high copy number, ease of amplification, moderate mutation rate, and maternal inheritance that avoids recombination—making it an ideal tool for inferring population history, dispersal routes, and initial patterns of genetic divergence [63,64]. However, the mitochondrial genome represents only a single genetic locus, and its inferences should be validated with more comprehensive nuclear genomic evidence [65]. Due to maternal inheritance bottlenecks, local selective pressures, and phylogenetic biases associated with low GC content, mitochondrial data may not fully reflect genome-wide population dynamics. Recent studies have shown that in insects, mitochondrial and nuclear genomes often exhibit topological discordance due to differences in GC composition [60], suggesting that the estimated divergence time of approximately 0.019 Ma and inferred demographic expansion in this study may be subject to bias. Moreover, key evolutionary processes such as hybridization, introgression, and male-mediated gene flow remain undetectable using mitochondrial markers alone. Future work should build upon this solid foundation by integrating nuclear genomic data (e.g., RAD-seq or whole-genome resequencing), landscape genomics, and functional validation approaches to elucidate the environmental drivers and molecular mechanisms underlying adaptive divergence, thereby advancing pest management from descriptive spatial zoning toward mechanism-informed, precision-based control strategies.

By systematically dissecting the population genetic structure of *R. pedestris* in soybean fields across China, this study provides “map-level” decision support for pest management. The southern–southwestern regions (Guangxi, Guizhou, Yunnan, Jiangxi, Fujian, and Anhui) exhibit the highest genetic diversity, representing a potential reservoir of resistance genes and a source of genetic variation; these areas should be designated as “priority pest management zones.” In contrast, populations in northern China—including Northeast (Liaoning, Jilin), North (Hebei, Shanxi, Shandong), and Northwest (Shaanxi, Gansu) subregions—generally exhibit lower genetic diversity, making them highly vulnerable to localized outbreaks if insecticide resistance rapidly escalates; thus, they should be classified as “priority monitoring zones for insecticide resistance”. This work offers a promising new paradigm for China’s soybean industry—shifting pest management from reactive responses toward proactive, design-based strategies.

## 5. Conclusions

In summary, this study, based on the analysis of mitochondrial gene (COI, COII, Cytb) concatenated sequences from 35 geographic populations in China, suggests that *R. pedestris* has relatively high genetic diversity across its geographic populations, with sequences showing a pronounced AT bias and more transitions than transversions. Both haplotype diversity (Hd) and nucleotide diversity (π) are moderately to highly variable, which may reflect a certain degree of environmental adaptability and potential for population differentiation. The pairwise genetic distances between populations range from 0.000 to 0.039, and the fixation index (FST) values range from −0.111 to 1.000. Notably, elevated FST values were observed between populations from the southwest and south regions compared to those from other areas, suggesting greater genetic differentiation in these comparisons. Phylogenetic analysis consistently resolved two major clades: Clade 1 (comprising populations from southern, southwestern, and parts of eastern China) and Clade 2 (including populations from central, northern, northwestern, northeastern, and parts of eastern China). Clade 2 displays a star-like topology, which could be consistent with a recent demographic expansion. When populations were grouped into three geographical regions—south, southwest, and others—AMOVA revealed that the majority of genetic variation (58.77%) was partitioned among groups, with a total *Fst* of 0.77014 (*p* < 0.001). Furthermore, comparisons of Nst and Gst indicated that Nst was significantly higher than Gst, suggesting a significant association between genetic divergence and geographic distance.

## Figures and Tables

**Table 1:** The nucleotide composition of the combined COI, COII, and Cytb gene sequences.

Codon	T	A	G	C
1st	42.39	44.61	2.70	10.30
2nd	33.52	32.56	20.56	13.36
3rd	40.08	21.02	17.79	21.12
Avg	38.67	32.73	13.68	14.92

**Table 2:** Gene composition and replacement frequency statistics of the COI, COII, and Cytb gene sequences.

Codon	ii	si	sv	R	TT	TC	TA	TG	CT	CC	CA	CG	AT	AC	AA	AG	GT	GC	GA	GG
Avg	2128	27	3	10	824	10	1	0	9	312	0	0	0	0	701	4	0	0	4	291
1st	698	19	3	7	298	7	1	0	6	67	0	0	0	0	317	3	0	0	3	16
2nd	712	7	0	0	238	2	0	0	3	93	0	0	0	0	233	1	0	0	1	147
3rd	718	1	0	0	288	0	0	0	0	152	0	0	0	0	151	0	0	0	0	128

Note: ii represents identical base sites; si represents the number of transitions, sv represents the number of transversions, and R represents si/sv.

**Table 3:** Genetic diversity indices for 35 populations of *R. pedestris* based on concatenated mitochondrial sequences (COI + COII + Cytb).

Code	Hap	Hd	π	K
DA	H1(1)H2(1)H3(5)H4(1)H5(1)H6(1)	0.7778	0.0022	4.7111
NN	H7(2)H8(6)H9(1)H10(1)	0.6444	0.0058	12.4667
GY	H3(2)H11(5)H12(1)H13(1)H14(1)	0.7556	0.0113	24.3778
ZY	H15(10)	0.0000	0.0000	0.0000
QL	H16(2)H17(1)H18(3)H19(1)H20(1)H21(1)H22(1)	0.9111	0.0066	14.2000
SM	H5(1)H13(1)H18(3)H23(2)H24(1)H25(1)H26(1)	0.9111	0.0056	12.1556
HZ	H18(8)H27(1)H28(1)	0.3778	0.0046	9.8222
LT	H29(3)H30(1)H31(1)H32(1)H33(1)H34(1)H35(1)H36(1)	0.9333	0.0144	30.9778
NC	H3(1)H37(1)H38(3)H39(1)H40(1)H41(1)H42(1)H43(1)	0.9333	0.0182	39.3111
GA	H44(1)H45(1)H46(1)H47(1)H48(1)H49(1)H50(1)H51(1)H52(1)H53(1)	1.0000	0.0095	20.4000
FZ	H54(3)H55(2)H56(1)H57(1)H58(1)H59(1)H60(1)	0.9111	0.0187	40.3556
HF	H18(3)H61(5)H62(1)H63(1)	0.7111	0.0187	40.4222
BJ	H55(6)H61(1)H64(1)H65(1)H66(1)	0.7556	0.0022	4.8222
AFJ	H67(1)H68(1)H69(1)H70(1)H71(1)H72(2)H73(1)H74(1)H75(1)	0.9778	0.0072	15.4889
FLJ	H76(5)H77(1)H78(1)H79(2)H80(1)	0.7556	0.0011	2.4222
BB	H18(1)H55(7)H81(2)	0.5111	0.0077	16.6444
ZK	H82(1)H83(3)H84(1)H85(5)	0.7111	0.0013	2.7111
SZ	H55(1)H73(5)H86(1)H87(1)H88(1)H89(1)	0.7778	0.0023	4.9778
XZ	H55(3)H90(2)H91(2)H92(1)H93(1)H94(1)	0.8889	0.0037	8.0222
SBJ	H95(3)H96(1)H97(1)H98(1)H99(1)H100(1)H101(1)H102(1)	0.9333	0.0035	7.6000
ZCY	H55(3)H103(1)H104(1)H105(1)H106(2)H107(1)H108(1)	0.9111	0.0022	4.8444
QJG	H109(3)H110(2)H111(1)H112(1)H113(1)H114(1)H115(1)	0.9111	0.0036	7.7333
YHW	H116(9)H117(1)	0.2000	0.0001	0.2000
QHB	H55(1)H118(1)H119(2)H120(1)H121(1)H122(1)H123(1)H124(1)H125(1)	0.9778	0.0043	9.2000
YA	H126(3)H127(1)H128(1)H129(1)H130(1)H131(2)H132(1)	0.9111	0.0015	3.2222
QY	H55(2)H133(5)H134(1)H135(1)H136(1)	0.7556	0.0021	4.4667
FY	H55(3)H94(1)H137(6)	0.6000	0.0025	5.3333
SJN	H55(1)H138(2)H139(1)H140(1)H141(1)H142(3)H143(1)	0.9111	0.0021	4.6222
JN	H144(1)H145(1)H146(1)H147(1)H148(4)H149(1)H150(1)	0.8667	0.0032	6.8444
CZ	H55(4)H96(1)H151(1)H152(1)H153(1)H154(1)H155(1)	0.8667	0.0018	3.7778
CD	H156(2)H157(5)H158(1)H159(1)H160(1)	0.7556	0.0022	4.8222
JZ	H55(2)H161(1)H162(1)H163(1)H164(1)H165(1)H166(1)H167(1)H168(1)	0.9778	0.0039	8.4667
KZ	H55(1)H169(1)H170(1)H171(1)H172(1)H173(1)H174(1)H175(1)H176(1)H177(1)H178(1)	1.0000	0.0041	8.9111
SY	H178(1)H179(9)	0.2000	0.0006	1.2000
JY	H73(3)H180(3)H181(1)H182(1)H183(1)H184(1)	0.8667	0.0027	5.8000

**Code**: population code; **Hap**: haplotype composition, with numbers in parentheses indicating the number of individuals sharing each haplotype (e.g., H1(1)H2(1) = two distinct haplotypes, each represented by one individual); **Hd**: haplotype diversity; **π**: nucleotide diversity; **K**: average number of pairwise nucleotide differences. Hd is zero in the Zunyi (ZY) population because all 10 individuals share the same haplotype (H15), resulting in no genetic variation within the sample.

**Table 4:** The analysis of molecular (AMOVA) of 35 populations of *R. pedestris*.

Source of Variation	df	Sum of Square	Variance Component	Percentage of Variation	Fixation Indices
Among groups	2	1799.14	14.42737 Va	58.77	FCT = 0.58774 ***
Within groups	32	1613.323	4.47738 Vb	18.24	FSC = 0.44243 ***
Within population	315	1777.4	5.64254 Vc	22.99	FST = 0.77014 ***

Note: *** *p <* 0.001.

**Table 5:** The Gst values (above diagonal) and Nst values (below diagonal) of pairwise difference among groups of 35 populations of *R. pedestris* of the COI, COII, and Cytb genes.

Group	Group 1	Group 2	Group 3
Group 1		0.05541	0.01951
Group 2	0.24098		0.01829
Group 3	0.71088	0.44819	

**Table 6:** Neutrality tests and mismatch distribution analyses for each group of *R. pedestris*.

Group	Tajima’s D	Fu’s Fs	SSD	Harpending’s Raggedness Index
Overall	−0.04157	−23.4844 *	0.01388	0.00247
Group 1	2.04583	4.23737	0.05836	0.08499
Group 2	2.4995	8.13761	0.03535	0.06776
Group 3	−1.20178	−23.64885 **	0.00463	0.00308

Note: ** *p* < 0.01, * *p* < 0.05.

## Data Availability

The original contributions presented in this study are included in the article/Supplementary Material. Further inquiries can be directed to the corresponding author.

## Supplementary Materials

The following supporting information can be downloaded at: https://www.mdpi.com/article/10.3390/insects17030337/s1, Figure S1: P distance among 35 populations of the COI gene; Figure S2: Fst values among 35 populations of COI gene; Figure S3: Nm values among 35 populations of COI gene; Figure S4: ML phylogenetic trees of the COI gene based on 35 populations of *R. pedestris*; Figure S5: BI phylogenetic trees of the COI gene based on 35 populations of *R. pedestris*; Figure S6: Haplotype network of the COI gene based on 35 populations of *R. pedestris*; Figure S7: P distance among 35 populations of the COII gene; Figure S8: Fst values among 35 populations of the COII gene; Figure S9: Nm values among 35 populations of the COII gene; Figure S10: ML phylogenetic trees of the COII gene based on 35 populations of *R. pedestris*; Figure S11: NJ phylogenetic trees of the COII gene based on 35 populations of *R. pedestris*; Figure S12: Haplotype network of the COII gene based on 35 populations of *R. pedestris*; Figure S13: P distance among 35 populations of the Cytb gene; Figure S14: Fst values among 35 populations of Cytb gene; Figure S15: Nm values among 35 populations of Cytb gene; Figure S16: ML phylogenetic trees of the Cytb gene based on 35 populations of *R. pedestris*; Figure S17: BI phylogenetic trees of the Cytb gene based on 35 populations of *R. pedestris*; Figure S18: Haplotype network based on 35 populations of *R. pedestris*; Table S1: Specimen data of different geographic populations of *R. pedestris*; Table S2: PCR primers; Table S3: The nucleotide sequences of the COI gene; Table S4: Gene composition and replacement frequency statistics of COI gene sequences; Table S5: Genetic diversity analysis of COI gene sequences; Table S6: The nucleotide sequences of the COII gene; Table S7: Gene composition and replacement frequency statistics of COII gene sequences; Table S8: Genetic diversity analysis of COII gene sequences; Table S9: The nucleotide of sequences of the COI gene; Table S10: Gene composition and replacement frequency statistics of COI gene sequences; Table S11: Genetic diversity analysis of Cytb gene sequences. Table S12: Comparison of genetic diversity parameters of three mitochondrial genes (COI, COII, Cytb) fragments and their combined sequences.
