# Supplementary material for: Mitogenomic Insight into the Population Genetic Diversity and Phylogeography of Soybean Stink Bug (Riptortus pedestris) in China
Source: Insects. 2026 Mar 19;17(3):337. doi: 10.3390/insects17030337 (PMC13026615; doi:10.3390/insects17030337)
Supplement: Supplementary file 1 [file insects-17-00337-s001.zip › insects-4080892-supplementary.pdf]

Supplementary Materials

Table S1. Specimen data of difference geographic populations of *R. pedestris*

	Location	Code	Coordinates	Elvation	Collecting Dates
Guangxi	Du'an	DA	23° 93'27.04"N,108° 10' 52.29"E	1048 m	2018.01
	Nanning	NN	22° 81'77.04"N,108° 36' 64.07"E	260m	2018.01
Guizhou	Guiyang	GY	26° 64'66.94"N,106° 62' 82.01"E	1140 m	2019.07
	Zunyi	ZY	27° 72'98.61"N,107° 03' 44.76"E	920m	2022.07
Yunnan	Qujing(Qilin)	QL	25° 52'41.30"N,103° 85' 43.32"E	1860 m	2018.08
	Songming	SM	25° 32'72.73"N,103° 04' 33.84"E	1910 m	2018.08
	Qujing(Huize)	HZ	26° 41'71.16"N,103° 29' 71.55"E	2120 m	2018.08
Jiangxi	Nanchang(Liantang)	LT	28° 54'45.46"N,115° 93' 48.78"E	26m	2019.07
	Nanchang	NC	28° 68'29.76"N,115° 85' 79.72"E	22m	2019.07
	Gao'an	GA	28° 33'51.70"N,115° 56' 31.25"E	25m	2019.07
Fujian	Fuzhou	FZ	26° 07'42.86"N,119° 29' 64.11"E	700m	2019.05

Anhui	Hefei	HF	31° 88'81.2"N,117° 24'60.19"E	30m	2018.09
	Bengbu(Baoji)	BJ	33° 16'41.51"N,117° 12'43.33"E	20m	2018.09
	Suzhou(YongqiaoDistrictFengjia)	AFJ	33° 62'63.95"N,116° 98'73.93"E	27m	2018.09
	Suzhou(Fuliji)	FLJ	33° 75'47.15"N,116° 97'72.79"E	35m	2018.09
	Bengbu	BB	33° 10'12.92"N,116° 87'23.36"E	23m	2018.09
Henan	Zhoukou	ZK	33° 76'22.85"N,114° 40'25.7"E	54m	2019.09
Jiangsu	Suzhou	SZ	31° 29'97.58"N,120° 58'52.94"E	10m	2019.08
	Xuzhou	XZ	31° 33'09.47"N,121° 07'00.34"E	29m	2018.09
Shaanxi	Baoji	SBJ	34° 36'28.62"N,107° 23'76.82"E	630m	2019.08
	Yan'an(Zhangcunyi)	ZCY	35° 89'84.82"N,109° 14'19.95"E	1390m	2022.08
	Yan'an(Qiaojiagou)	QJG	35° 92'40.45"N,109° 30'79.56"E	1390m	2022.08
	Yan'an(Yanhewan)	YHW	36° 76'58.08"N,109° 36'52.07"E	1400m	2022.08
	Yan'an(Qinghuabian)	QHB	36° 78'99.26"N,109° 65'40.85"E	1100m	2022.08
	Yan'an	YA	36° 65'01.09"N,109° 49'46.8"E	1200m	2019.08

Gansu	Qingyang	QY	35° 70'94.59"N,107° 64'34.33"E	1800 m	2019.08
Shanxi	Fenyang	FY	37° 26'14.93"N,111° 77'08.34"E	1400 m	2018.09
Shandong	Jinan	SJN	35° 32'06.16"N,115° 49'68.26"E	140 m	2018.09
	Jining	JN	35° 41'51.17"N,116° 58'71.16"E	37m	2018.09
Hebei	Cangzhou	CZ	38° 30'46.76"N,116° 83'87.15"E	12m	2018.09
	Chengde	CD	40° 95'29.42"N,117° 96'27.49"E	350 m	2019.09
Tianjin	Jizhou	JZ	40° 04'65.44"N,117° 40'84.32"E	1100 m	2020.09
Liaoning	Chaoyang(Kazuo)	KZ	41° 07'35.09"N,120° 06'80.26"E	340 m	2022.08
	Shenyang	SY	41° 68'33.99"N,123° 47'33.95"E	450 m	2022.08
Jilin	Baishan(Jingyu)	JY	42° 38'89.02"N,126° 81'32.62"E	570 m	2017.08

Table S2 PCR primers

Gene	Primer name	Primer sequence（5'-3'）
COI	P1	TTTACACCTTTAGACTTGC
	P2	CTAAAGTTCATAATGTAGCAGGT
COII	R1	AATTTACAAGATGCTATTTCCCCTCT
	R2	TCCTGGTGTTGCATCGATTT
Cytb	F1	TTGTAAACATTTCTGAAGGACC

F2	CGTGCTCCAATTCAAGTT

3.1 Genetic Diversity Analysis Based on Mitochondrial COI Gene Sequences

3.1.1 Base Composition of COI Gene Sequences

Analysis of 350 mitochondrial COI gene sequences (844 bp in length) from 35 geographic populations of *R. pedestris* revealed a strong A+T bias in base composition. The average nucleotide frequencies were T = 38.64%, A = 30.39%, G = 15.66%, and C = 15.31%, resulting in an overall A+T content of 69.03% and G+C content of 30.97% (Table S3). Among the three codon positions, the third position exhibited the highest A+T content (66.55%), followed by the first (75.85%), while the second position was more balanced (45.13%). A total of 73 variable sites were identified, including 65 parsimony-informative sites and 8 singleton sites. Nucleotide substitution analysis showed 11 transitions (si) and only 1 transversion (sv), yielding a transition/transversion ratio (R) of 9 (Table S4). Notably, no transitions or transversions were detected at the second and third codon positions, indicating strong evolutionary conservation at these sites. Among the 16 possible base pair combinations, homologous pairs TT and AA were most abundant, followed by GG and CC; heterologous combinations were rare.

Table S3 The nucleotide of COI gene sequences

Codon	T	A	G	C
1st	45.36	45.92	1.13	7.59
2nd	27.12	29.89	27.76	15.24
3rd	43.43	15.30	18.15	23.12
Avg	38.64	30.39	15.66	15.31

Table S4 Genes composition and replacement frequence statistics of COI gene sequences

Codon	ii	si	sv	R	TT	TC	TA	TG	CT	CC	CA	CG	AT	AC	AA	AG	GT	GC	GA	GG
1st	271	10	1	9	124	4	1	0	3	17	0	0	0	0	128	1	0	0	1	2
2nd	281	0	0	15	76	0	0	0	0	43	0	0	0	0	84	0	0	0	0	78
3rd	281	0	0	0	122	0	0	0	0	65	0	0	0	0	43	0	0	0	0	51
Avg	832	11	1	9	322	5	1	0	3	125	0	0	0	0	255	1	0	0	1	131

Note: ii represents identical base sites; si represents the number of transitions, sv represents the number of transversions, and R represents si/sv.

3.1.2 Genetic Diversity of COI Gene Sequences

The overall genetic diversity of *R. pedestris* based on COI sequences was high. A total of 56 haplotypes (Hap) were identified across the 35 populations, with haplotype diversity (*Hd*) of 0.9113, nucleotide diversity (π) of 0.01389, sequence diversity (*K*) of 11.72, and 73 polymorphic sites (*S*) (Table S5). Significant variation in genetic diversity was observed among populations. The Zunyi (ZY, Guizhou), Zhoukou (ZK, Henan), and Shenyang (SY, Liaoning) populations exhibited zero haplotype diversity, indicating high genetic uniformity within these populations. In contrast, the highest *Hd* (0.9111) was observed in the Kazuo (KZ, Liaoning) population. Populations with high nucleotide diversity were primarily distributed in East China, including Nanchang (NC, Jiangxi; π = 0.0165), Fuzhou (FZ, Fujian; π = 0.0171), and Hefei (HF, Anhui; π = 0.0172), all of which also showed *K* values exceeding 10.

Table S5 Genetic diversity analysis of COI gene sequences

Code	Hap	Hd	π	K
DA	H13(8)H14(1)H15(1)	0.3778	0.0005	0.4000
NN	H13(1)H35(9)	0.2000	0.0036	3.0000
GY	H7(6)H13(2)H18(2)	0.6222	0.0023	1.9556
ZY	H56(10)	0.0000	0.0000	0.0000
QL	H7(4)H13(2)H15(2)H18(2)	0.8000	0.0027	2.3111
SM	H7(7)H13(3)	0.4667	0.0017	1.4000
HZ	H7(8)H18(2)	0.3556	0.0017	1.4222
LT	H28(3)H29(1)H30(3)H31(1)H32(1)H33(1)	0.8667	0.0152	12.8222
NC	H1(1)H7(1)H13(2)H34(6)	0.6444	0.0165	13.9111
GA	H7(2)H50(5)H51(2)H52(1)	0.7333	0.0127	10.6889
FZ	H1(3)H15(1)H18(5)H19(1)	0.7111	0.0171	14.4000
HF	H5(5)H6(1)H7(3)H8(1)	0.7111	0.0172	14.5111
BJ	H1(8)H5(1)H9(1)	0.3778	0.0012	1.0000
AFJ	H1(2)H2(2)H3(3)H4(2)H5(1)	0.8667	0.0049	4.1556
FLJ	H11(9)H16(1)	0.2000	0.0005	0.4000
BB	H1(7)H7(1)H27(2)	0.5111	0.0073	6.1778
ZK	H1(10)	0.0000	0.0000	0.0000
SZ	H1(1)H4(7)H46(1)H47(1)	0.5333	0.0030	2.5333
XZ	H1(3)H5(2)H17(2)H48(3)	0.8222	0.0038	3.2222
SBJ	H1(4)H2(2)H4(3)H45(1)	0.7778	0.0034	2.8889
ZCY	H1(5)H49(1)H53(1)H54(1)H55(2)	0.7556	0.0036	3.0222
QJG	H40(1)H42(5)H43(2)H44(2)	0.7333	0.0045	3.7778
YHW	H1(9)H49(1)	0.2000	0.0002	0.2000
QHB	H1(2)H11(1)H36(2)H37(1)H38(1)H39(1)H40(1)H41(1)	0.9556	0.0043	3.6000
YA	H43(9)H45(1)	0.2000	0.0007	0.6000

QY	H1(2)H11(8)	0.3556	0.0013	1.0667
FY	H1(3)H4(6)H17(1)	0.6000	0.0035	2.9333
SJN	H1(2)H17(2)H20(6)	0.6222	0.0030	2.4889
JN	H1(1)H11(6)H20(2)H21(1)	0.6444	0.0014	1.1556
CZ	H1(7)H2(1)H11(1)H12(1)	0.5333	0.0012	1.0000
CD	H1(5)H10(4)H11(1)	0.6444	0.0032	2.7333
JZ	H1(2)H11(2)H20(3)H22(1)H23(2)	0.8667	0.0032	2.6889
KZ	H1(3)H10(1)H11(1)H12(1)H24(2)H25(1)H26(1)	0.9111	0.0038	3.2222
SY	H11(10)	0.0000	0.0000	0.0000
JY	H1(3)H2(3)H4(4)	0.7333	0.0037	3.1333

3.1.3 Inter-population Genetic Distances Based on COI Sequences

Pairwise genetic distances (p-distances) among the 35 populations ranged from 0.000 to 0.039 (Figure S1). Larger genetic distances (0.003–0.037) were observed between populations from Southwest and South China (e.g., Guangxi, Guizhou, Yunnan) and those from Central, East, North, Northwest, and Northeast China. The greatest distances were found between Zunyi (ZY, Guizhou) and Du'an (DA, Guangxi) or Nanning (NN, Guangxi), reaching 0.033 and 0.039, respectively, indicating substantial genetic differentiation. In contrast, most populations from Central, East, North, Northwest, and Northeast China showed very low genetic distances (0.000–0.006), suggesting a relatively homogeneous genetic structure in these regions.

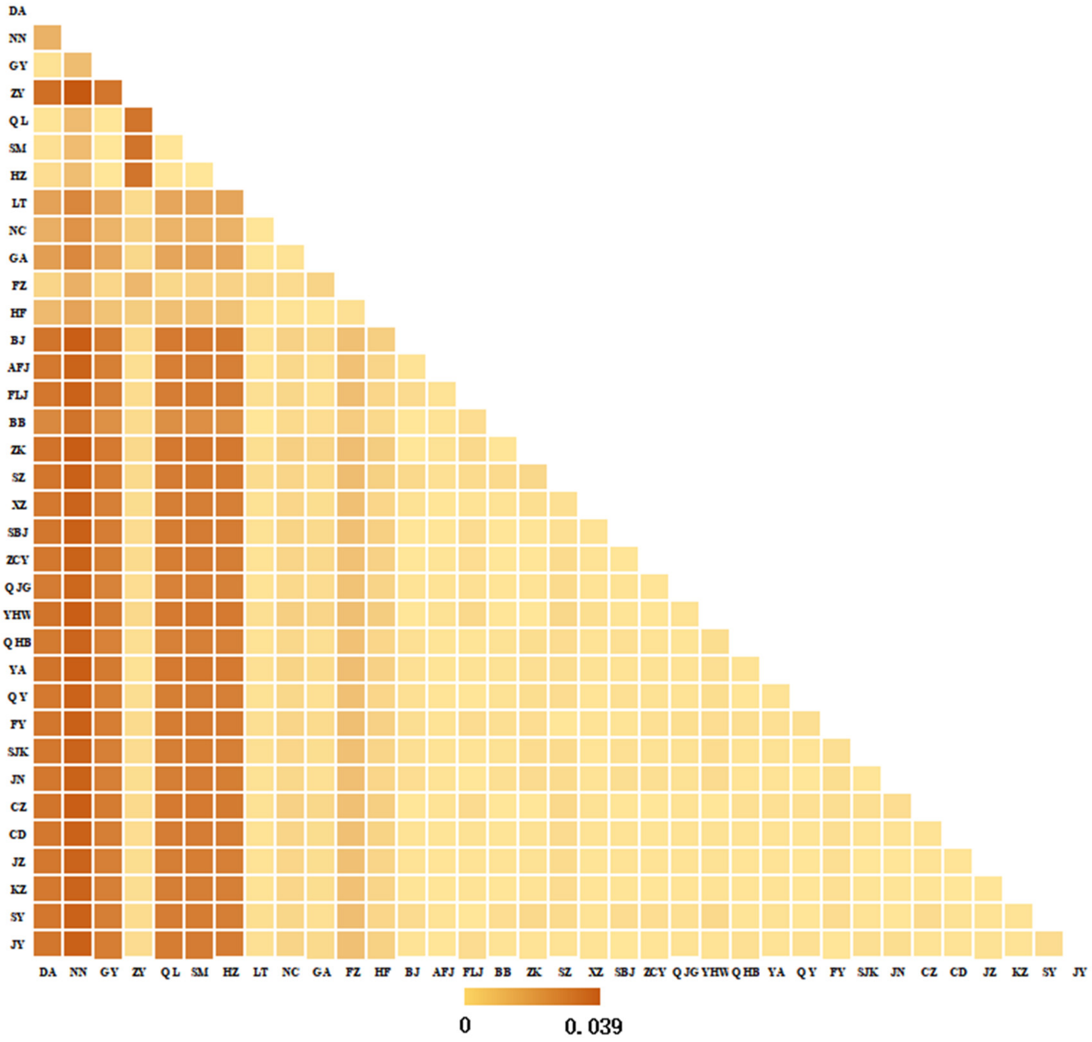

Figure S1 The P distance among 35 populations of COI gene

3.1.4 Gene Flow and Population Differentiation Based on COI Sequences

The pairwise *Fst* values among populations ranged from –0.062 to 0.993 (Figure S2). High *Fst* values (>0.280) were consistently observed between Sout hwest/South China populations and those from other regions, indicating strong genetic differentiation. For example, *Fst* between Du'an (DA, Guangxi) and mu ltiple northern populations approached or exceeded 0.9. However, negative *Fst* values were observed between Guiyang (GY, Guizhou) and other Southwest po pulations (e.g., QL, SM, HZ), suggesting potential gene flow or sampling artifa cts. Corresponding gene flow (*Nm*) estimates ranged from –101.056 to 100.152 (Figure S3). Most *Nm* values between Southwest/South China and other regions

were <1, indicating limited gene flow. In contrast, *Nm* >1 was common among populations in Central, East, North, Northwest, and Northeast China, with many exceeding 4, suggesting frequent gene exchange and near-panmixia in these regions.

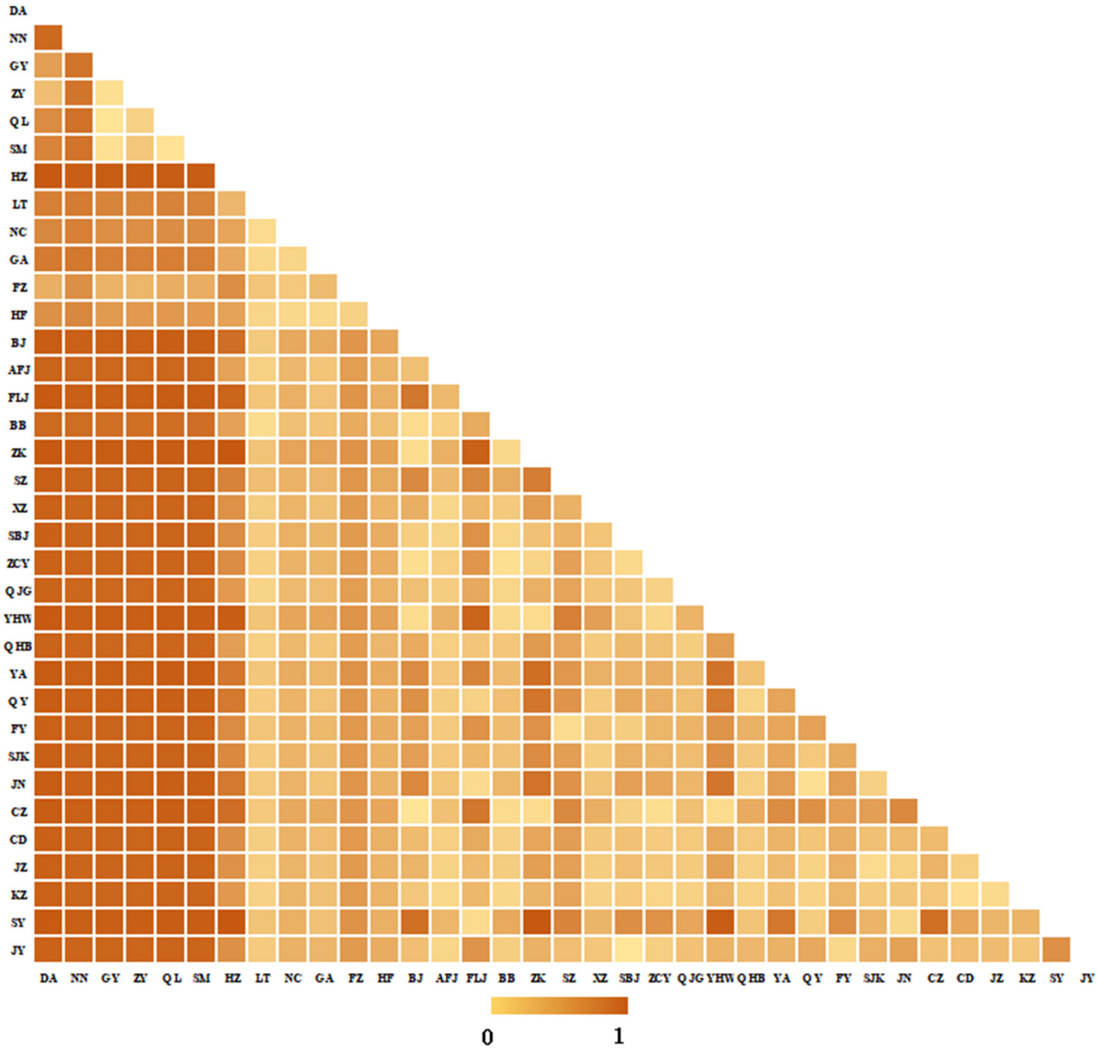

Figure S2 The Fst Values among 35 populations of COI gene

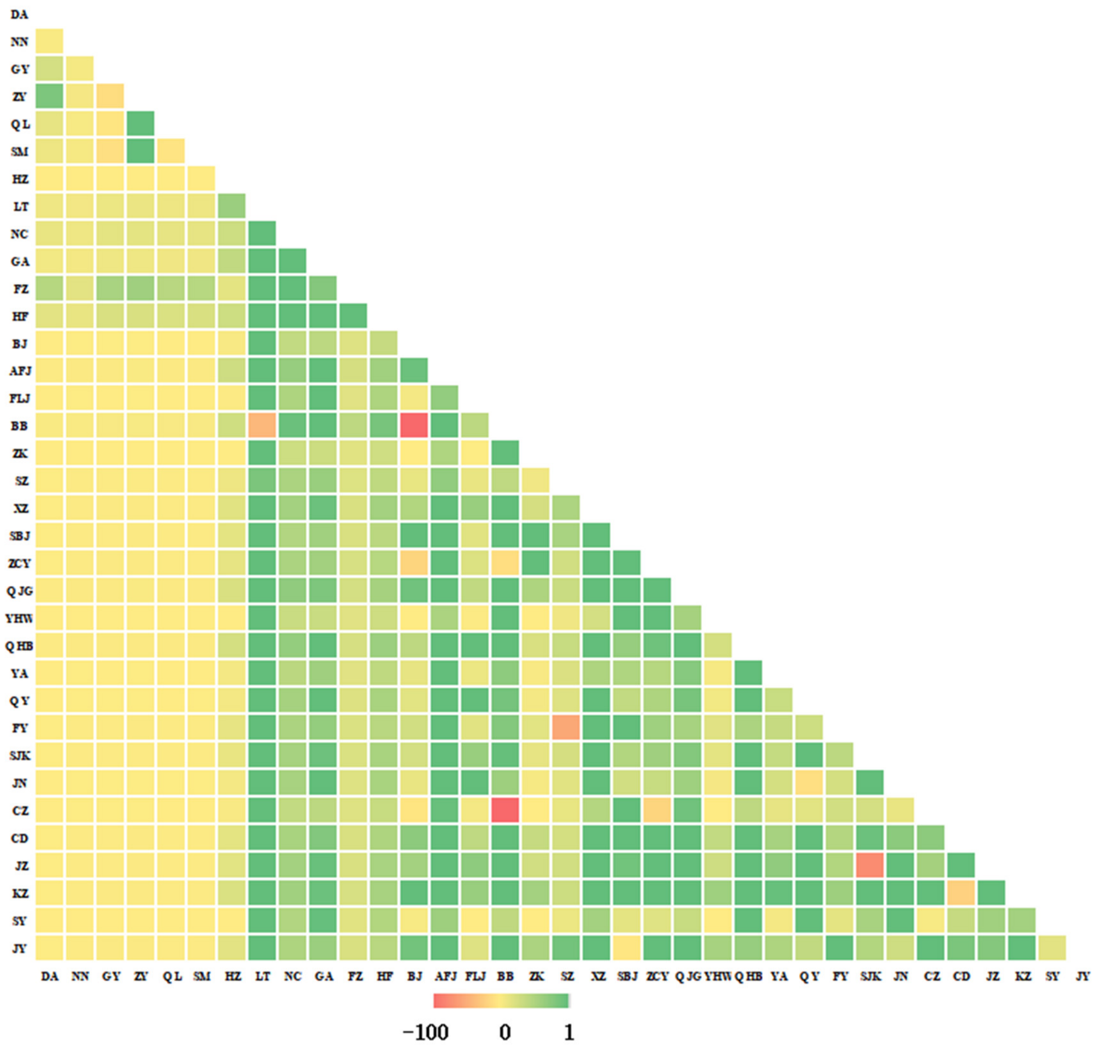

Figure S3 The Nm values among 35 populations of COI gene

3.1.5 Phylogenetic and Haplotype Network Analyses Based on COI Sequences

Maximum Likelihood (ML) and Bayesian Inference (BI) trees based on COI sequences yielded highly congruent topologies, both dividing *R. pedestris* into two major clades (Clade 1 and Clade 2) (Figures S4 and S5). Clade 1 included 9 haplotypes, primarily composed of populations from Southwest and South China: all samples from Guiyang (GY, Guizhou), Yunnan (QL, SM, HZ), and Guangxi (DA, NN), along with subsets from Jiangxi (LT, NC, GA), Fujian (FZ), and Anhui (HF, BJ). The Zunyi (ZY) haplotype formed a distinct subclade within this group. Clade 2 contained 47 haplotypes, encompassing all popul

ations from Central, East, North, Northwest, and Northeast China, as well as some individuals from Zunyi (ZY).

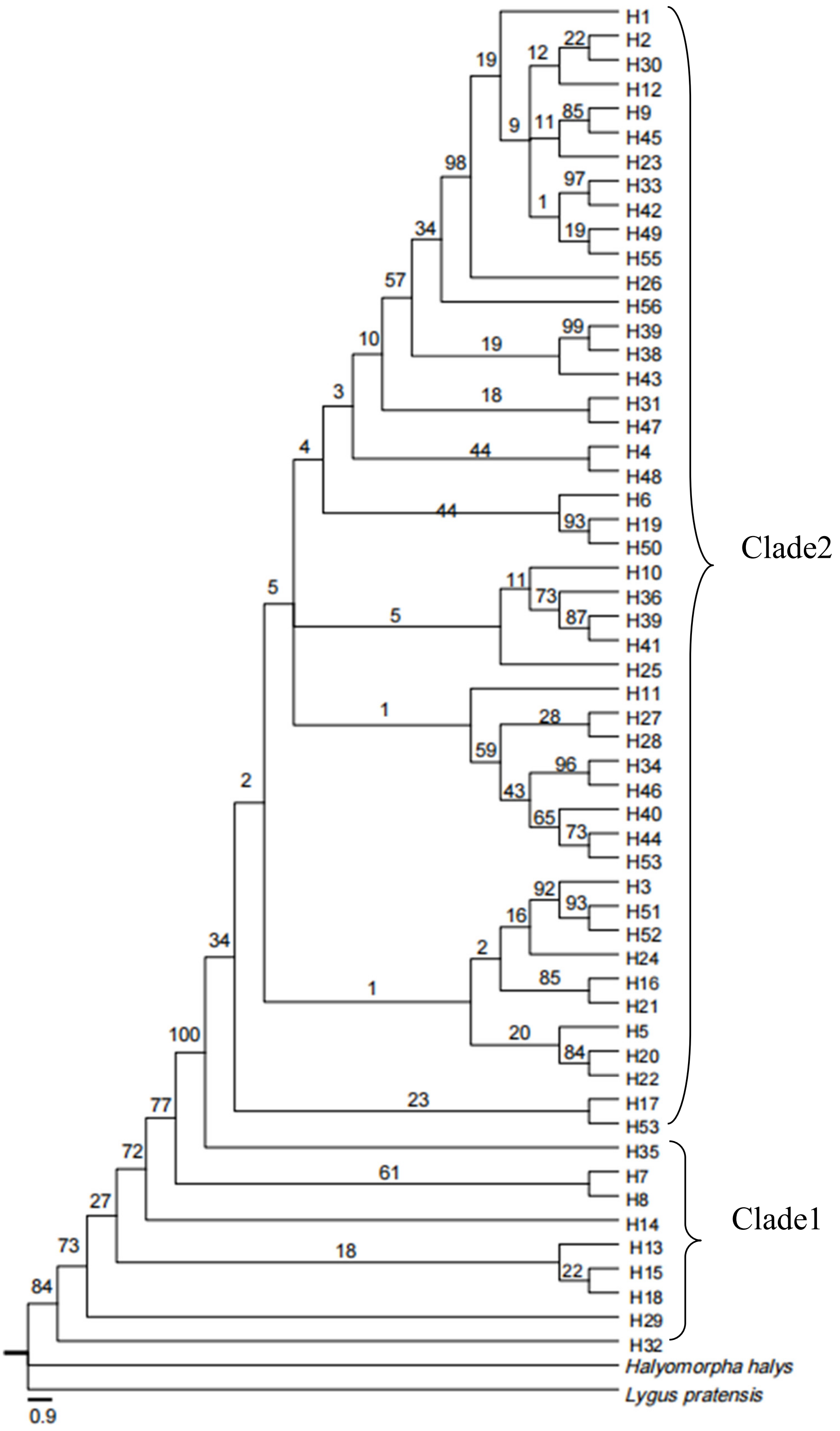

Figure S4 COI gene ML Phylogenetic trees based on 35 populations of *R. pedestris*

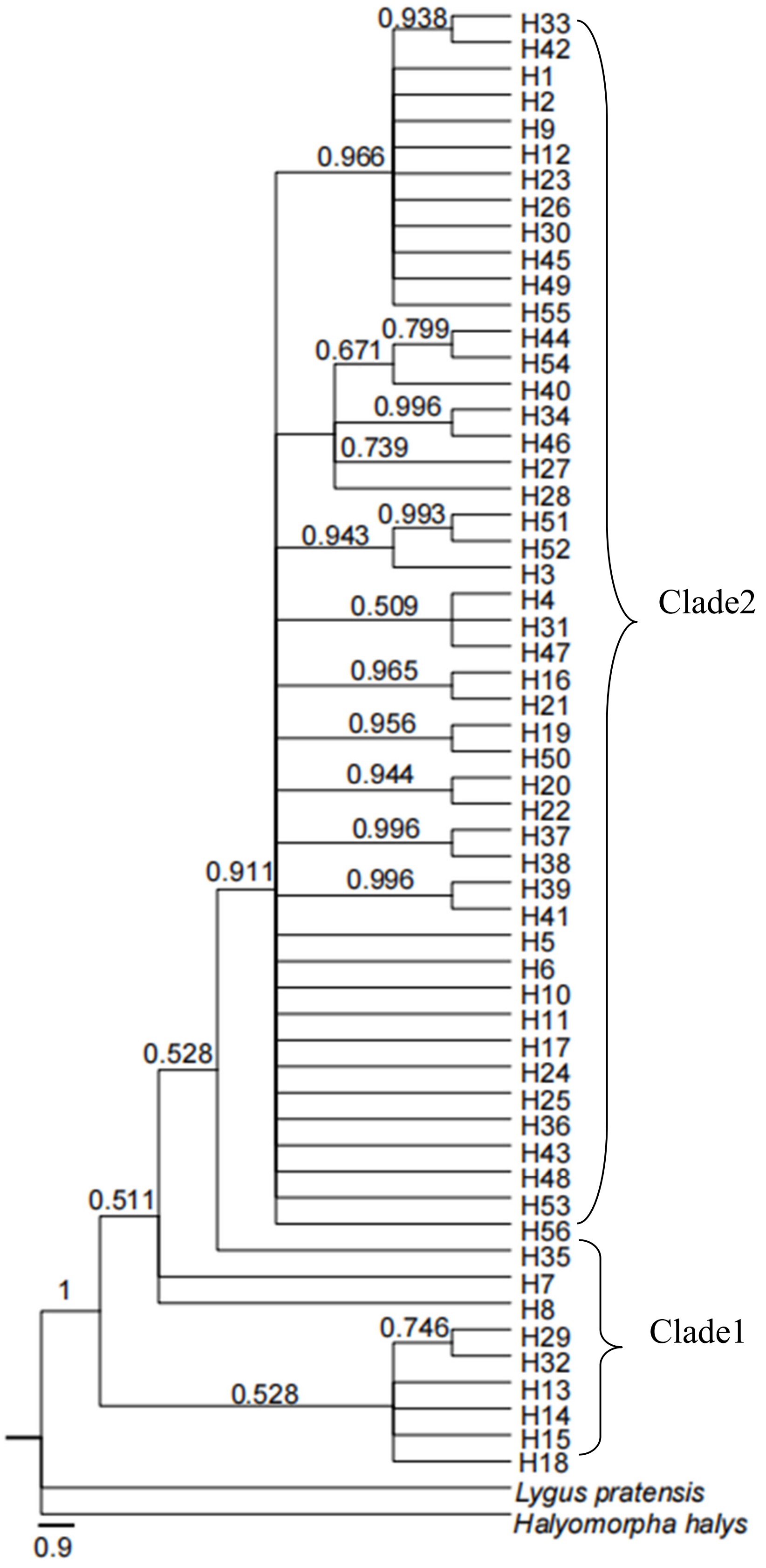

Figure S5 COI gene BI Phylogenetic trees based on 35 populations of *R. pedestris*

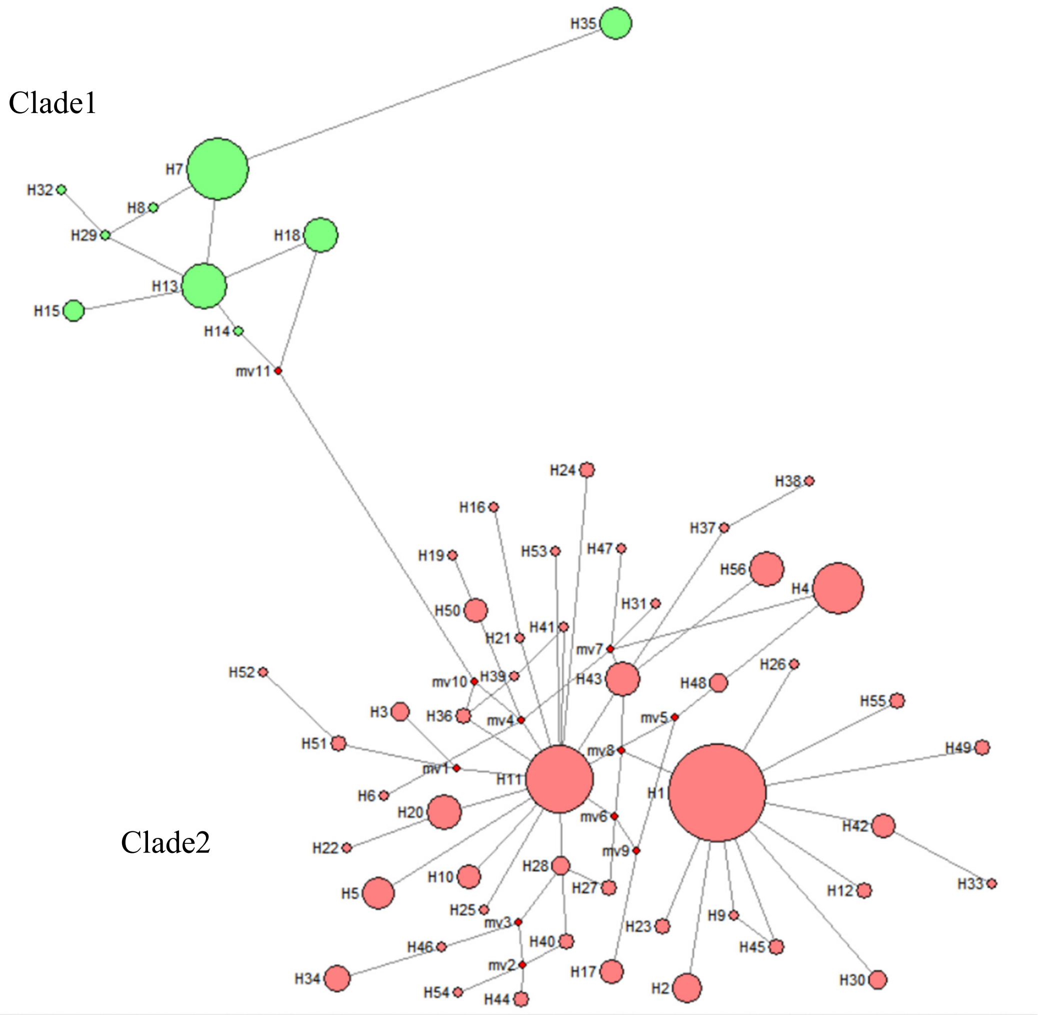

Figure S6 Haplotype network of COI gene based on 35 populations of *R. pedestris*

The haplotype network (Figure 2.6) corroborated this pattern, with 11 med ian vectors introduced. Clades 1 and 2 were clearly separated. In Clade 1, hap lotype H13 occupied the central position and was shared by six populations, su ggesting it as the ancestral haplotype. Haplotype H35 (Nanning, NN) was dista ntly located, indicating genetic uniqueness. In Clade 2, haplotype H1 was centr al and shared by 21 populations, likely representing the ancestral lineage. The nested, star-like distribution of haplotypes in Clade 2 suggests a recent rapid p opulation expansion and diversification.

3.2 Genetic Diversity Analysis Based on Mitochondrial COII Gene Sequences

3.2.1 Base Composition of COII Gene Sequences

Analysis of 350 mitochondrial COII gene sequences (473 bp) from *R. pedestris* revealed a strong A+T bias. The average contents of T, A, G, and C were 39.32%, 35.35%, 11.16%, and 14.17%, respectively, resulting in an A+T content of 74.67% and a G+C content of 25.33% (Table S6), a bias even stronger than that of the COI gene. The sequence contained 28 variable sites, including 24 parsimony-informative sites. Base substitution analysis revealed a notable feature: no transversions (sv=0) were detected across the entire sequence; transitions (si) occurred only at the first and second codon positions, with very few at the second position (Table S7), indicating that the second and third codon positions of the COII gene are highly conserved during evolution. Among nucleotide combinations, TT and AA were the most abundant, far exceeding other combinations.

Table S6 The nucleotide of COII gene sequences

Codon	T	A	G	C
1st	46.08	43.73	3.73	6.45
2nd	27.95	31.72	22.08	18.26
3rd	43.95	30.57	7.64	17.83
Avg	39.32	35.35	11.16	14.17

Table S7 Genes composition and replacement frequence statistics of COII gene sequences

Codon	ii	si	sv	R	TT	TC	TA	TG	CT	CC	CA	CG	AT	AC	AA	AG	GT	GC	GA	GG
1st	153	5	0	0	71	1	0	0	2	9	0	0	0	0	68	1	0	0	1	5
2nd	157	1	0	0	44	0	0	0	0	29	0	0	0	0	50	0	0	0	0	35
3rd	157	0	0	0	69	0	0	0	0	28	0	0	0	0	48	0	0	0	0	12
Avg	468	5	0	0	184	2	0	0	2	65	0	0	0	0	166	1	0	0	1	52

Note: ii represents identical base sites; si represents the number of transitions, sv represents the number of transversions, and R represents si/sv.

3.2.2 Genetic Diversity of COII Gene Sequences

Genetic diversity analysis of the COII gene indicated high overall genetic variation in *R. pedestris*. A total of 20 haplotypes were identified across 35 po pulations, with haplotype diversity (Hd) of 0.8142, nucleotide diversity (π) of 0. 01159, sequence diversity (K) of 5.47, and 28 polymorphic sites (S) (Table S 8). Genetic diversity varied significantly among populations. Populations from Z unyi (ZY) in Guizhou, Yanhewan (YHW) in Shaanxi, Jinan (SJN), and Shenya ng (SY) in Liaoning showed zero haplotype diversity. The highest haplotype di versity was observed in Qinghuabian (QHB) in Shaanxi (Hd = 0.9333). Only t he Hefei (HF) population in Anhui had sequence diversity exceeding 10 (K = 10.0000), and it also had the highest nucleotide diversity (π = 0.0212) among all populations. Populations with high nucleotide diversity were mainly distribut ed in southwestern, southern, and eastern China, such as Nanning (NN) in Gua ngxi, Qujing (QL) and Songming (SM) in Yunnan, Nanchang (NC) in Jiangxi, Fuzhou (FZ) in Fujian, and Hefei (HF) and Anfujing (AFJ) in Anhui.

Table S8 genetic diversity analysis of COII gene sequences

Code	Hap	Hd	π	K
DA	H1(9)H7(1)	0.2000	0.0059	2.8000
NN	H1(6)H7(2)H15(2)	0.6222	0.0158	7.4667
GY	H1(3)H3(5)H4(2)	0.6889	0.0150	7.0889
ZY	H20(10)	0.0000	0.0000	0.0000
QL	H1(5)H4(4)H7(1)	0.6444	0.0169	7.9556
SM	H1(5)H4(4)H7(1)	0.6444	0.0169	7.9556
HZ	H1(9)H7(1)	0.2000	0.0059	2.8000
LT	H1(1)H2(2)H3(3)H14(4)	0.7778	0.0098	4.6000
NC	H1(6)H3(1)H4(3)	0.6000	0.0162	7.6667
GA	H2(5)H4(2)H18(3)	0.6889	0.0022	1.0222
FZ	H1(5)H3(4)H4(1)	0.6444	0.0176	8.3111
HF	H1(5)H2(5)	0.5556	0.0212	10.0000
BJ	H2(1)H3(9)	0.2000	0.0013	0.6000

AFJ	H1(4)H2(1)H3(4)H4(1)	0.7333	0.0183	8.6222
FLJ	H4(2)H5(8)	0.3556	0.0015	0.7111
BB	H1(1)H3(7)H13(2)	0.5111	0.0069	3.2667
ZK	H2(1)H3(3)H6(5)H8(1)	0.7111	0.0041	1.9111
SZ	H3(9)H8(1)	0.2000	0.0004	0.2000
XZ	H3(3)H5(1)H6(2)H8(4)	0.7778	0.0029	1.3556
SBJ	H3(5)H5(3)H6(2)	0.6889	0.0019	0.9111
ZCY	H3(7)H5(1)H9(1)H19(1)	0.5333	0.0013	0.6000
QJG	H3(6)H6(4)	0.5333	0.0023	1.0667
YHW	H3(10)	0.0000	0.0000	0.0000
QHB	H2(2)H3(1)H5(2)H6(1)H8(2)H16(1)H17(1)	0.9333	0.0055	2.6000
YA	H2(5)H3(4)H6(1)	0.6444	0.0044	2.0667
QY	H3(2)H6(8)	0.3556	0.0015	0.7111
FY	H3(3)H5(6)H4(1)	0.6000	0.0014	0.6667
SJN	H3(10)	0.0000	0.0000	0.0000
JN	H2(2)H3(8)	0.3556	0.0023	1.0667
CZ	H3(8)H5(2)	0.3556	0.0008	0.3556
CD	H3(1)H5(2)H6(7)	0.5111	0.0014	0.6667
JZ	H2(4)H3(3)H9(3)	0.7333	0.0044	2.0667
KZ	H2(4)H3(3)H10(1)H11(1)H12(1)	0.8000	0.0051	2.4000
SY	H9(10)	0.0000	0.0000	0.0000
JY	H2(2)H3(8)	0.7333	0.0044	2.0667

3.2.3 Inter-population Genetic Distance of COII Gene Sequences

The genetic distance (p-distance) among the 35 geographic populations bas ed on COII sequences ranged from -0.001 to 0.028 (Figure S7). Populations fr om Du'an (DA) in Guangxi and Huize (HZ) in Yunnan generally showed great er genetic distances from other populations. Notably, despite the close geograph ic proximity of Zunyi (ZY) in Guizhou to Du'an (DA) in Guangxi, their geneti c distance reached 0.028, indicating significant genetic differentiation. Overall, populations that were geographically distant (e.g., Guangxi vs. Northeast China) also exhibited greater genetic distances. In contrast, most populations in centra

l, eastern, northern, northwestern, and northeastern China showed small genetic distances, ranging from -0.001 to 0.014.

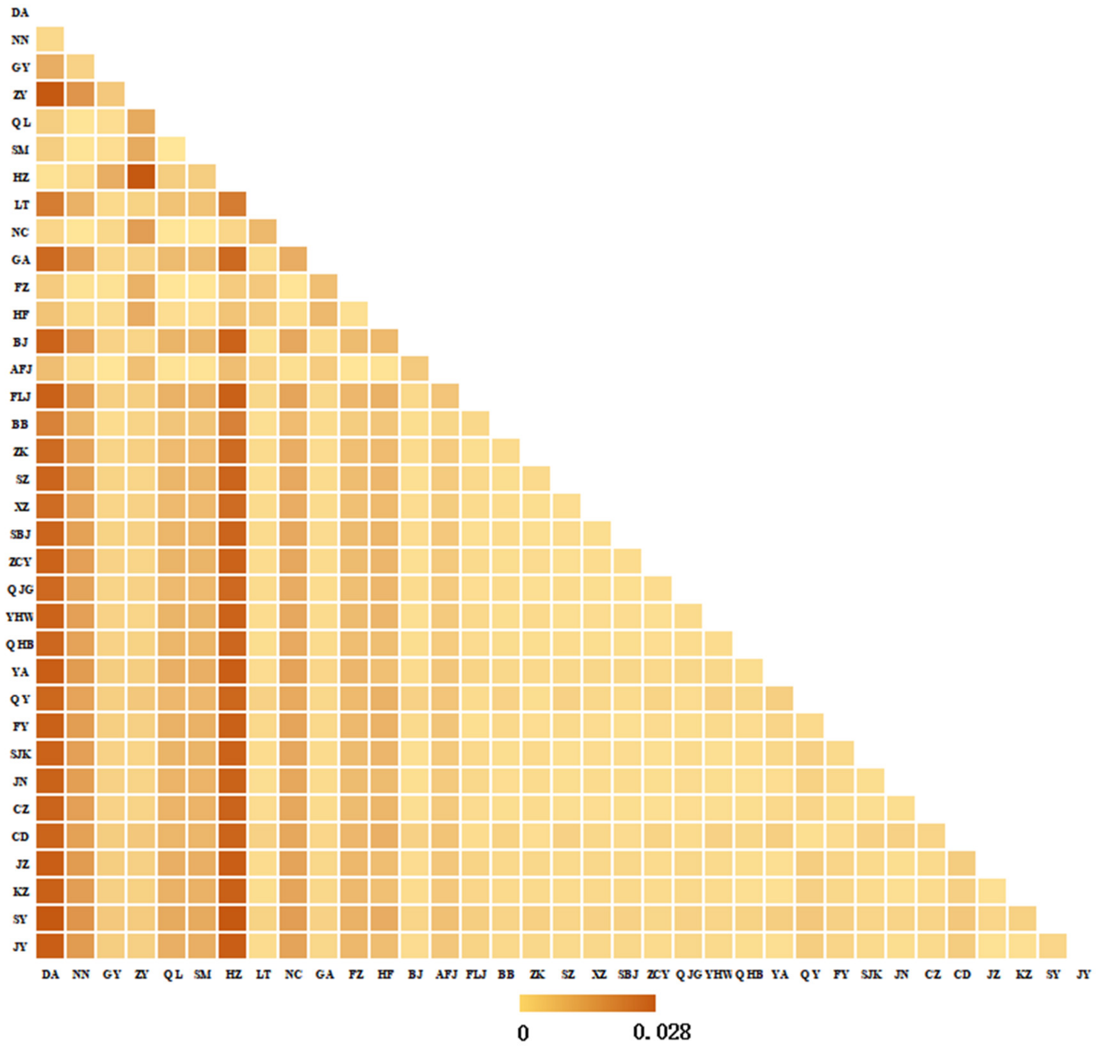

Figure S7 The P distance among 35 populations of COII gene

3.2.4 Gene Flow and Population Differentiation of COII Gene Sequences

The population genetic differentiation index (Fst) ranged from -0.111 to 1. 000 (Figure S8). High Fst values were generally observed between populations in Du'an (DA) in Guangxi and Huize (HZ) in Yunnan and those in other regio ns, indicating significant genetic differentiation. However, the Fst value between Du'an (DA) and Songming (SM) in Yunnan was -0.111, suggesting extremely high genetic similarity. Fst values between Huize (HZ) and Yanhewan (YHW) in Shaanxi, Jinan (SJN), and Shenyang (SY) reached 1.000, indicating complete

genetic differentiation. The calculated gene flow (Nm) ranged from -34.356 to 26.748 (Figure 2.9). The absolute Nm values between some populations in so uthwestern, southern, and eastern China were greater than 1, indicating some le vel of gene flow. In contrast, among populations in central, eastern, northern, n orthwestern, and northeastern China, absolute Nm values were often greater tha n 4, indicating frequent gene exchange.

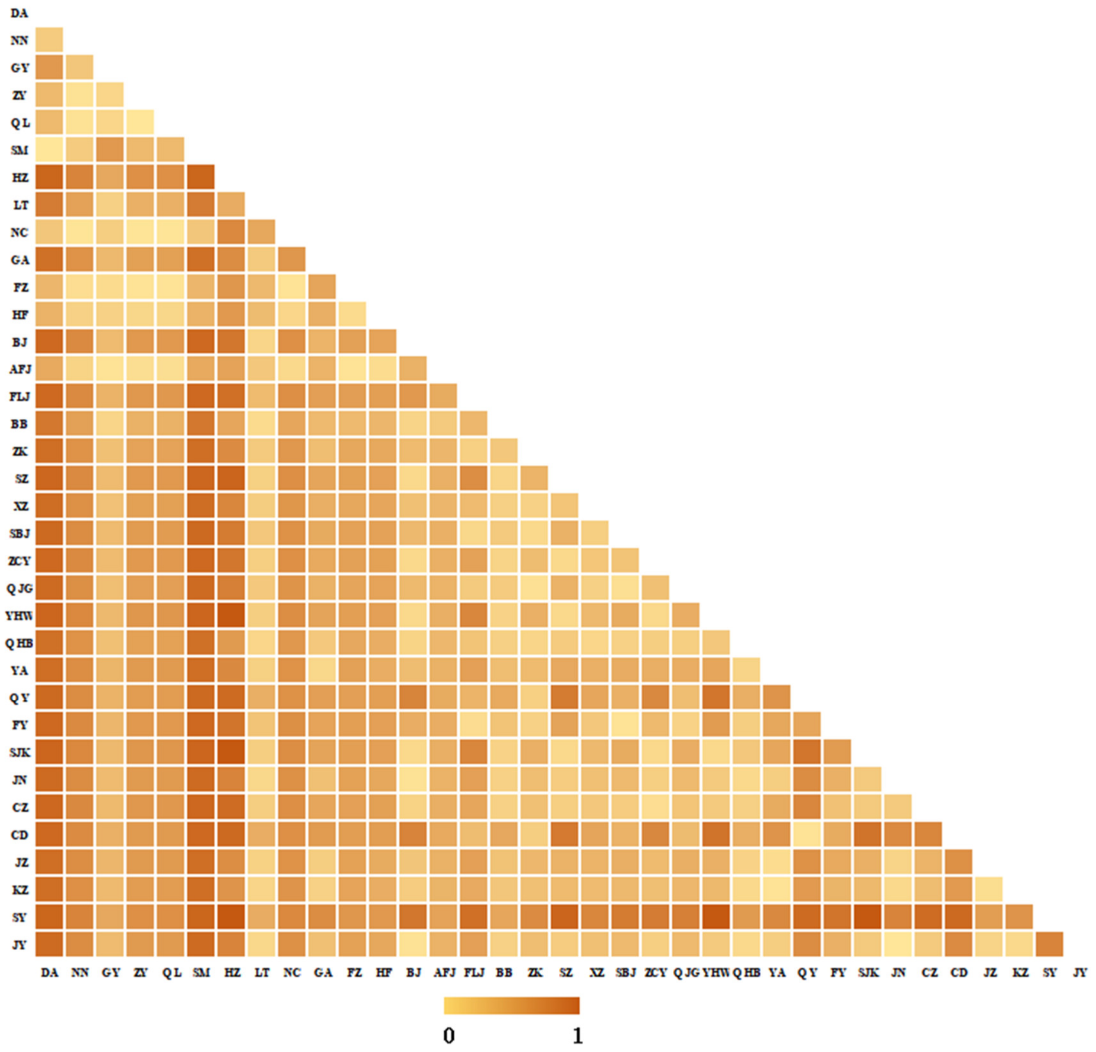

Figure S8 The Fst Values among 35 populations of COII gene

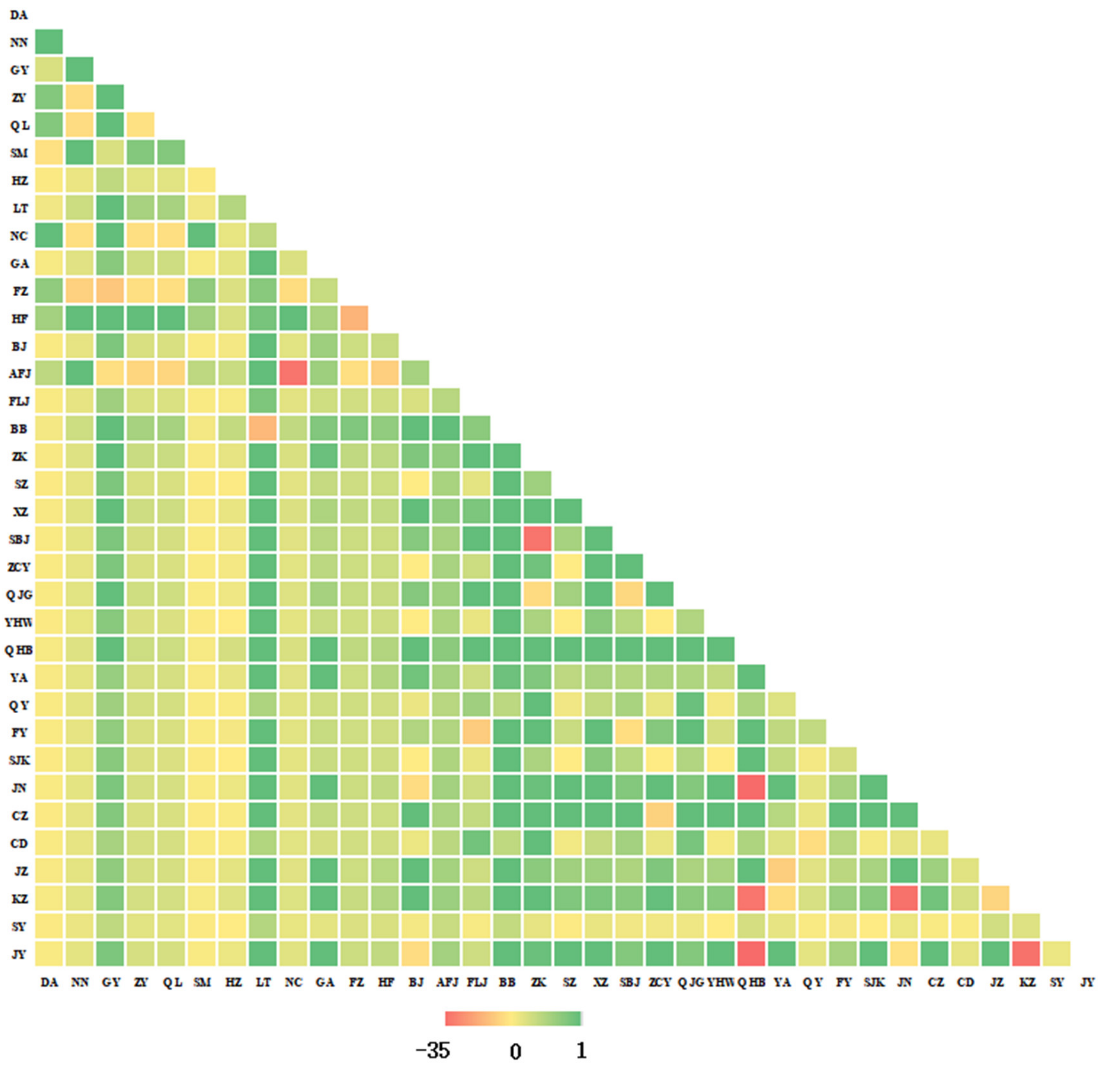

Figure S9 The Nm values among 35 populations of COII gene

3.2.5 Phylogenetic and Haplotype Network Analysis of COII Gene Sequenc es

The maximum likelihood (ML) and neighbor-joining (NJ) trees based on C OII sequences showed highly consistent topologies, both dividing *R. pedestris* i nto two main clades (Figure S10, Figure S11). Clade 1 contained 5 haplotypes, primarily composed of populations from southwestern and southern China, incl uding all samples from Guiyang (GY) in Guizhou, Qujing (QL), Songming (S M), and Huize (HZ) in Yunnan, and Du'an (DA) and Nanning (NN) in Guang xi, as well as partial samples from Liantang (LT), Nanchang (NC), and Gao’an (GA) in Jiangxi, Fuzhou (FZ) in Fujian, and Hefei (HF), Fuliangjiang (FLJ),

and Baoji (BB) in Anhui. The haplotype from Zunyi (ZY) in Guizhou was ind ependent of this clade. Clade 2 contained 15 haplotypes, encompassing all pop ulations from central, northern, northwestern, and northeastern China, as well as some from eastern and southwestern China. Due to the short sequence length, Bayesian inference (BI) failed to clearly resolve the geographic differentiation p attern. The haplotype network (Figure S12) also supported the existence of two main clades. Clade 1 included 5 haplotypes, with central haplotype H4 shared by 8 populations, suggesting it as the ancestral haplotype; H1 was the most widely distributed within this clade. Clade 2 included 15 haplotypes, with centr al haplotype H3 widely shared by 26 populations, representing the ancestral ha plotype of this clade. Its star-like structure suggests high genetic connectivity a nd recent population expansion. Populations from some areas in eastern and so uthwestern China (e.g., Jiangxi, Fujian, Anhui, and Zunyi in Guizhou) were dis tributed in both clades.

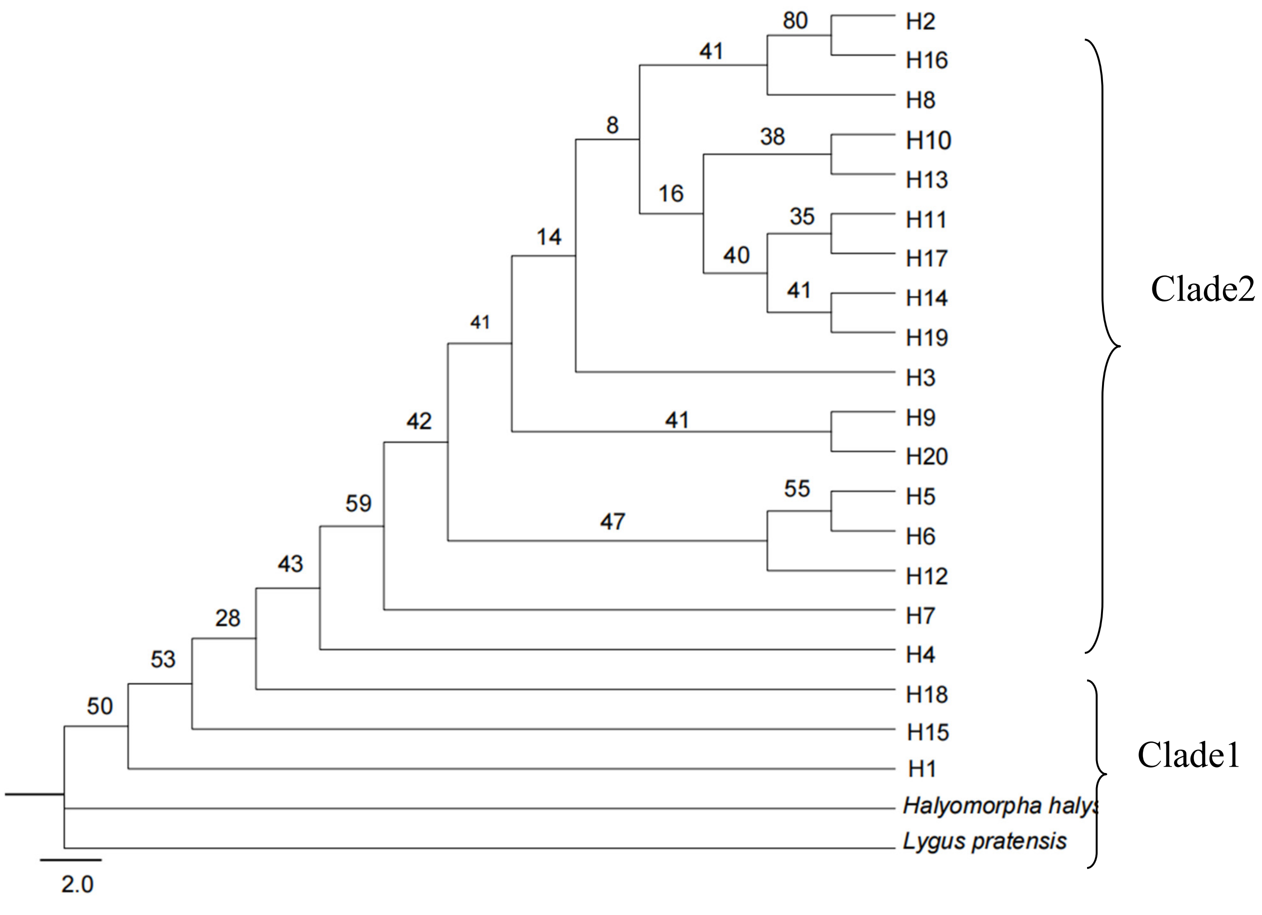

Figure S10 COII gene ML Phylogenetic trees based on 35 populations of *R. pedestris*

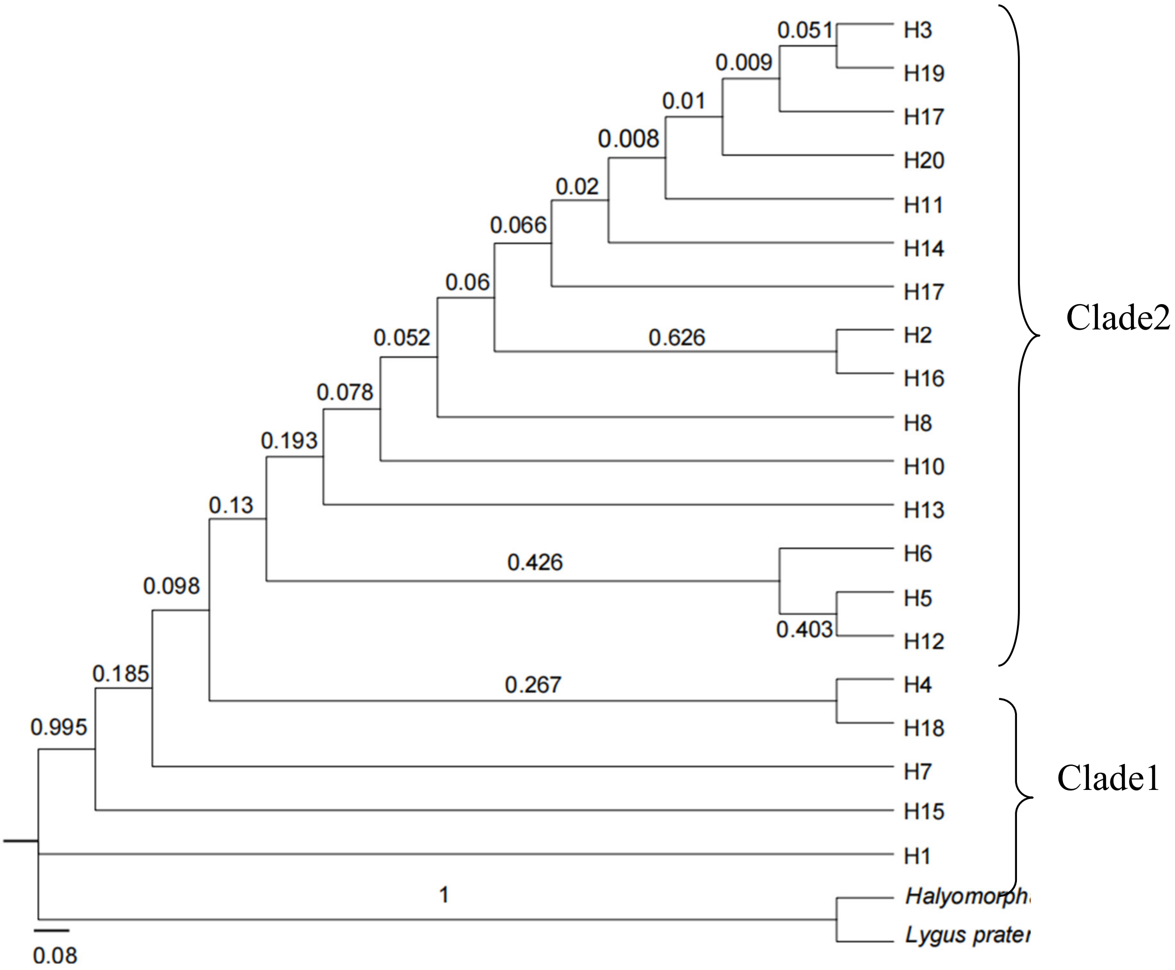

Figure S11 COII gene NJ Phylogenetic trees based on 35 populations of *R. pedestris*

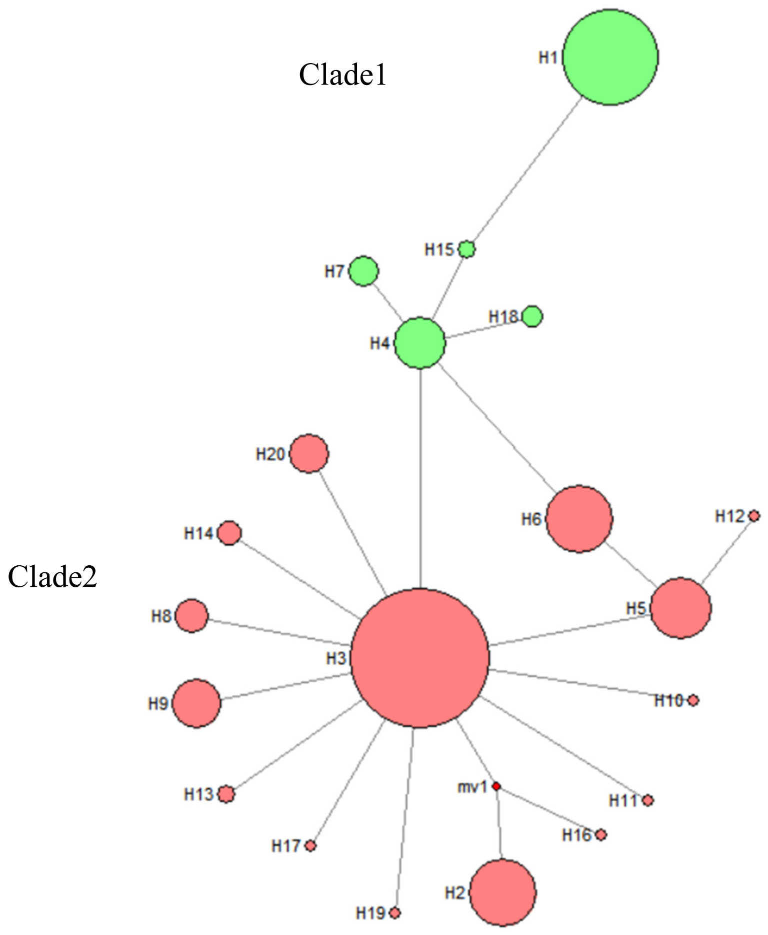

Figure S12 Haplotype network of COII gene based on 35 populations of *R. pedestris*

3.3 Genetic Diversity Analysis Based on Mitochondrial Cytb Gene Sequenc es

3.3.1 Base Composition of Cytb Gene Sequences

Analysis of 350 mitochondrial Cytb gene sequences (841 bp) from *R. ped estris* revealed a significant A+T bias. The average contents of T, A, G, and C were 38.32%, 33.62%, 13.11%, and 14.95%, respectively, resulting in an A+ T content of 71.94% and a G+C content of 28.06% (Table S9). Among the th ree codon positions, the third position had the highest A+T content (64.28%), f ollowed by the first (79.67%), while the second was more balanced (45.13%). The sequence contained 93 variable sites, including 84 parsimony-informative si tes. Base substitution analysis showed that the number of transitions (si = 11)

was much higher than transversions (sv = 1), with a transition/transversion rati o (R) of 8 (Table 2-12). No transversions occurred at the second codon positio n, and no transitions or transversions occurred at the second and third position s, indicating high evolutionary conservation at these sites. Among the 16 nucle otide combinations, TT and AA were the most abundant, followed by GG and CC, with others less common.

Table S9 The nucleotide of COI gene sequences

Codon	T	A	G	C
1st	38.53	51.14	1.52	8.81
2nd	32.86	29.89	27.76	15.36
3rd	43.57	20.71	15.00	20.71
Avg	38.32	33.62	13.11	14.95

Table S10 genes composition and replacement frequence statistics of COI gene sequences

Codon	ii	si	sv	R	TT	TC	TA	TG	CT	CC	CA	CG	AT	AC	AA	AG	GT	GC	GA	GG
		1								12										10
Avg	828	1	1	8	318	4	0	0	4	2	0	0	0	0	280	2	0	0	2	8
1st	270	9	1	7	105	3	0	0	3	22	0	0	0	0	141	2	0	0	2	2
2nd	278	2	0	37	91	1	0	0	1	42	0	0	0	0	81	0	0	0	0	64
3rd	280	0	0	0	122	0	0	0	0	58	0	0	0	0	58	0	0	0	0	42

Note: ii represents identical base sites; si represents the number of transitions, sv represent s the number of transversions, and R represents si/sv.

3.3.2 Genetic Diversity of Cytb Gene Sequences

Genetic diversity analysis of the Cytb gene indicated extremely high overa ll genetic diversity in *R. pedestris*. A total of 74 haplotypes were identified acr oss 35 populations, with haplotype diversity (Hd) of 0.92558, nucleotide diversi ty (π) of 0.01492, sequence diversity (K) of 12.55, and 93 polymorphic sites (S) (Table S11), the highest among the three genes. Genetic diversity varied si gnificantly among populations. The Zunyi (ZY) in Guizhou and Yanhewan (YH W) in Shaanxi populations had zero haplotype diversity. The highest haplotype

diversity was observed in Qinghuabian (QHB) in Shaanxi and Kazuo (KZ) in Liaoning, both reaching 0.9111. Populations with sequence diversity exceeding 10 included Guiyang (GY, K=15.33) in Guizhou, Liantang (LT, K=13.56) in Jia ngxi, Nanchang (NC, K=17.73) in Jiangxi, Fuzhou (FZ, K=17.64) in Fujian, an d Hefei (HF, K=15.91) in Anhui, all of which also had high nucleotide diversi ty (π>0.018), primarily distributed in eastern China.

Table S11 Genetic diversity analysis of Cytb gene sequences

Code	Hap	Hd	π	K
DA	H7(7)H14(1)H15(1)H16(1)	0.5333	0.0018	1.5111
NN	H46(9)H47(1)	0.2000	0.0024	2.0000
GY	H1(6)H7(3)H27(1)	0.6000	0.0182	15.3333
ZY	H74(10)	0.0000	0.0000	0.0000
QL	H7(1)H8(5)H22(3)H55(1)	0.7111	0.0047	3.9333
SM	H7(1)H8(5)H16(1)H47(2)H59(1)	0.7556	0.0033	2.8000
HZ	H1(1)H8(9)	0.2000	0.0067	5.6000
LT	H1(2)H6(2)H38(4)H39(1)H40(1)	0.8222	0.0161	13.5556
NC	H7(1)H8(1)H41(4)H42(1)H43(1)H44(1)H45(1)	0.8667	0.0211	17.7333
GA	H67(4)H68(2)H69(2)H70(1)H71(1)	0.8222	0.0103	8.6889
FZ	H1(3)H22(3)H23(1)H24(1)H25(1)H26(1)	0.8667	0.0210	17.6444
HF	H6(5)H7(1)H8(4)	0.6444	0.0189	15.9111
BJ	H1(7)H6(1)H8(1)H9(1)	0.5333	0.0082	6.8667
AFJ	H1(3)H2(1)H3(1)H4(3)H5(2)	0.8444	0.0032	2.7111
FLJ	H17(6)H18(1)H19(1)H20(2)	0.6444	0.0016	1.3111
BB	H1(7)H8(1)H37(2)	0.5111	0.0086	7.2000
ZK	H10(9)H28(1)	0.2000	0.0010	0.8000
SZ	H1(2)H5(6)H61(1)H62(1)	0.6444	0.0027	2.2444
XZ	H1(4)H5(3)H6(2)H21(1)	0.7778	0.0041	3.4444
SBJ	H1(2)H6(5)H9(1)H17(2)	0.7333	0.0045	3.8000
ZCY	H1(4)H11(2)H17(1)H72(1)H73(1)	0.8222	0.0015	1.2222
QJG	H1(5)H52(2)H53(2)H54(1)	0.7333	0.0034	2.8889
YHW	H66(10)	0.0000	0.0000	0.0000
QHB	H1(2)H48(1)H49(2)H50(2)H51(2)H52(1)	0.9111	0.0036	3.0000
YA	H63(7)H64(2)H65(1)	0.5111	0.0007	0.5556

QY	H1(2)H6(1)H56(5)H57(1)H58(1)	0.7556	0.0032	2.6889
FY	H1(3)H5(6)H21(1)	0.6000	0.0021	1.7333
SJN	H1(2)H5(5)H9(1)H17(2)	0.7333	0.0025	2.1333
JN	H5(4)H6(1)H21(1)H29(1)H30(1)H31(1)H32(1)	0.8667	0.0055	4.6222
CZ	H1(6)H6(1)H11(1)H12(1)H13(1)	0.6667	0.0029	2.4222
CD	H1(8)H10(2)	0.3556	0.0017	1.4222
JZ	H1(4)H6(1)H10(2)H28(2)H29(1)	0.8222	0.0044	3.7111
KZ	H1(3)H10(2)H11(1)H33(1)H34(1)H35(1)H36(1)	0.9111	0.0039	3.2889
SY	H10(1)H60(9)	0.2000	0.0014	1.2000
JY	H1(1)H5(9)	0.2000	0.0007	0.6000

3.3.3 Inter-population Genetic Distance of Cytb Gene Sequences

The genetic distance (p-distance) among the 35 geographic populations bas ed on Cytb sequences ranged from 0.000 to 0.037 (Figure S13). Populations fr om Yunnan and Guangxi showed greater genetic distances from those in centra l, eastern, northern, northwestern, and northeastern China, ranging from 0.004 t o 0.037. Notably, Zunyi (ZY) in Guizhou exhibited greater genetic distances fr om geographically nearby populations in Yunnan and Guangxi (0.028–0.037), w hile its distances to geographically distant populations in central, northern, nort hwestern, and northeastern China were less than 0.01. In contrast, most populat ions in central, eastern, northern, northwestern, and northeastern China showed very small genetic distances, ranging from 0.001 to 0.013.

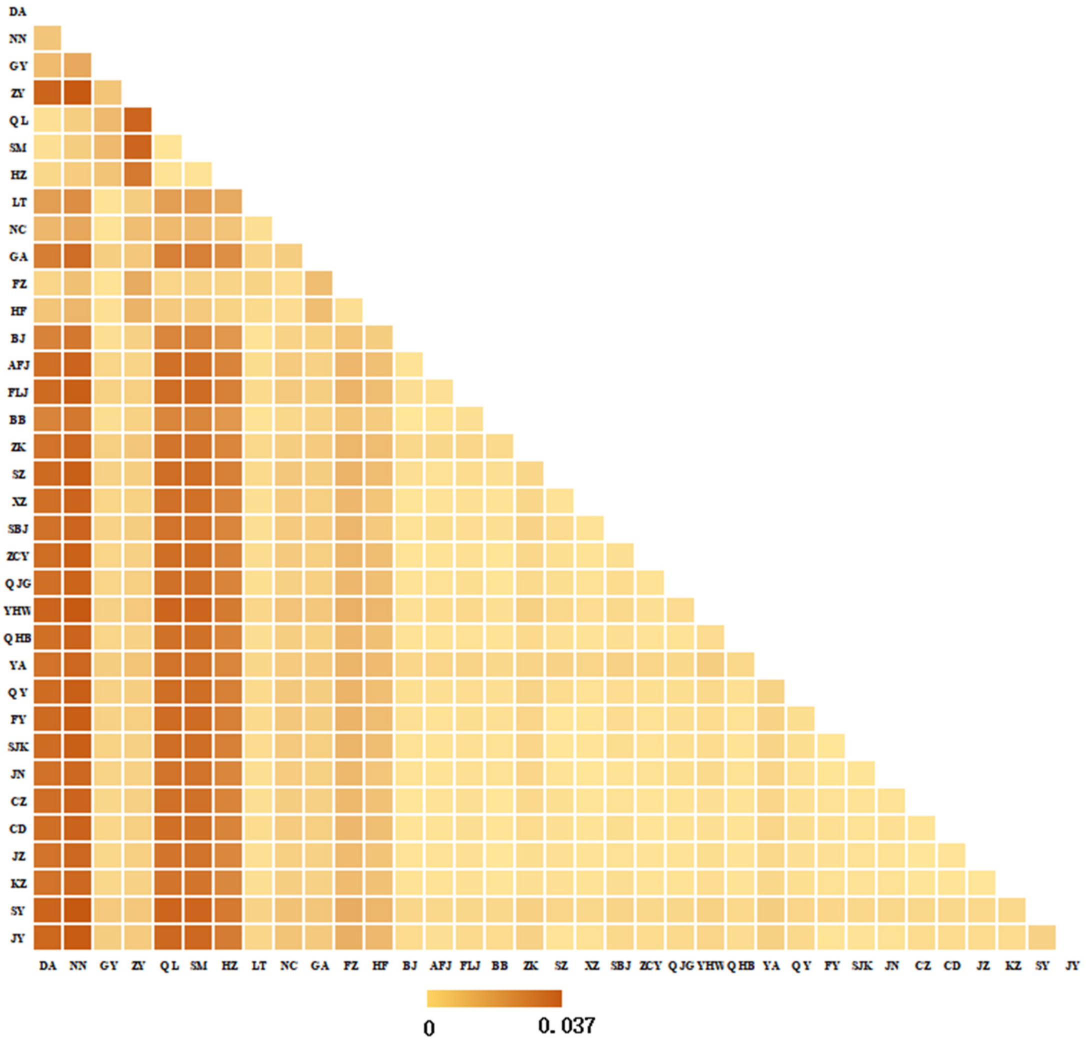

Figure S13 The P distance among 35 populations of Cytb gene

3.3.4 Gene Flow and Population Differentiation of Cytb Gene Sequences

The population genetic differentiation index (Fst) ranged from -0.099 to 1.000 (Figure S14). High Fst values were generally observed between populations in southwestern and southern China and those in other regions, indicating significant genetic differentiation. Populations from Zunyi (ZY) in Guizhou, Zhoukou (ZK) in Henan, and Yanhewan (YHW) in Shaanxi were particularly distinct, with Fst between ZY and YHW reaching 1.000, indicating complete differentiation. However, the Fst value between Guiyang (GY) in Guizhou and Fuzhou (FZ) in Fujian was negative, suggesting high genetic similarity. The calculated gene flow (Nm) ranged from -107.546 to 237.845 (Figure S15). Nm values bet

ween populations in Guangxi and Yunnan and those in other regions were mos tly less than 1, indicating restricted gene flow. In contrast, among most populat ions in central, eastern, northern, northwestern, and northeastern China, absolute Nm values were greater than 1, and some exceeded 4, indicating frequent gen e exchange.

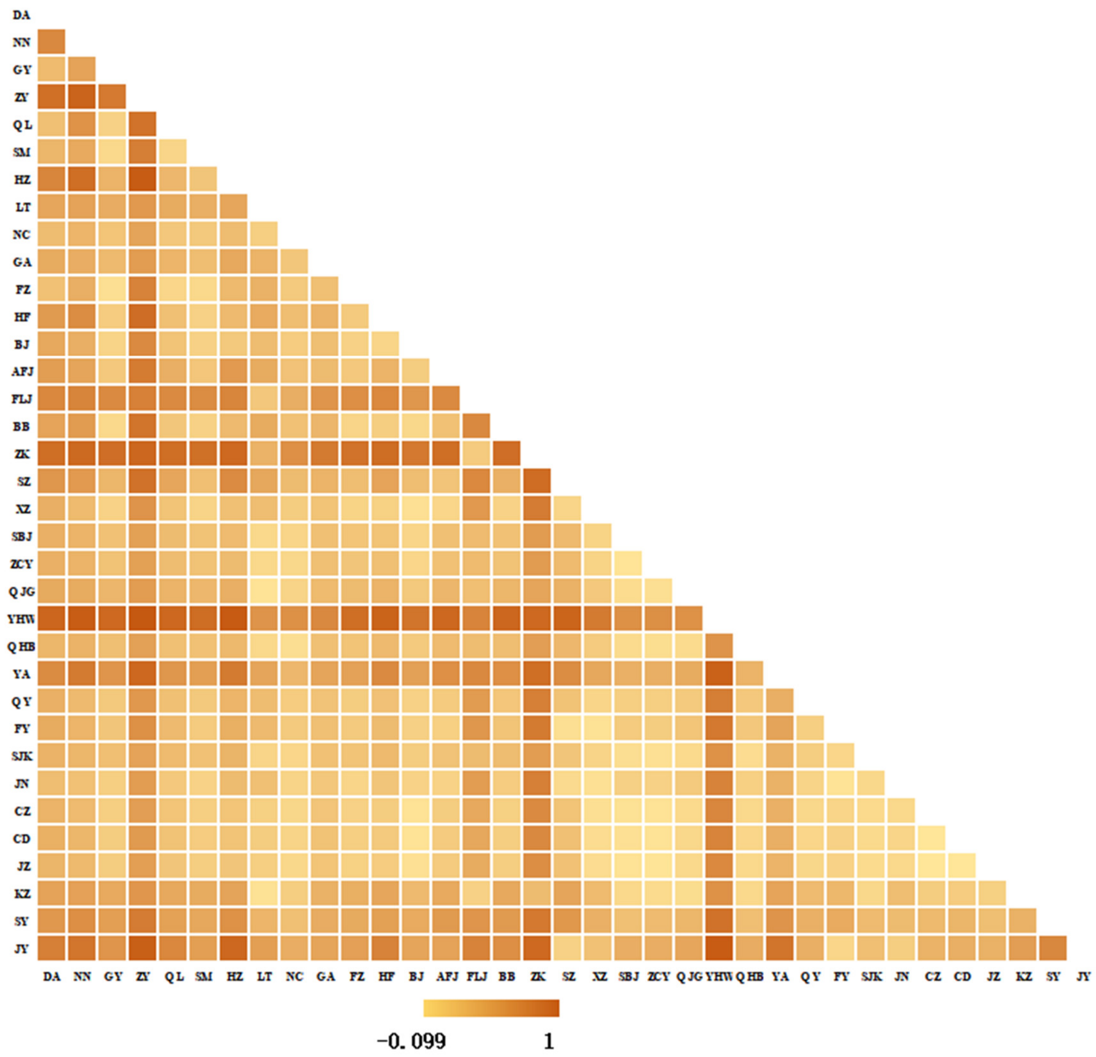

Figure S14 The Fst Values among 35 populations of Cytb gene

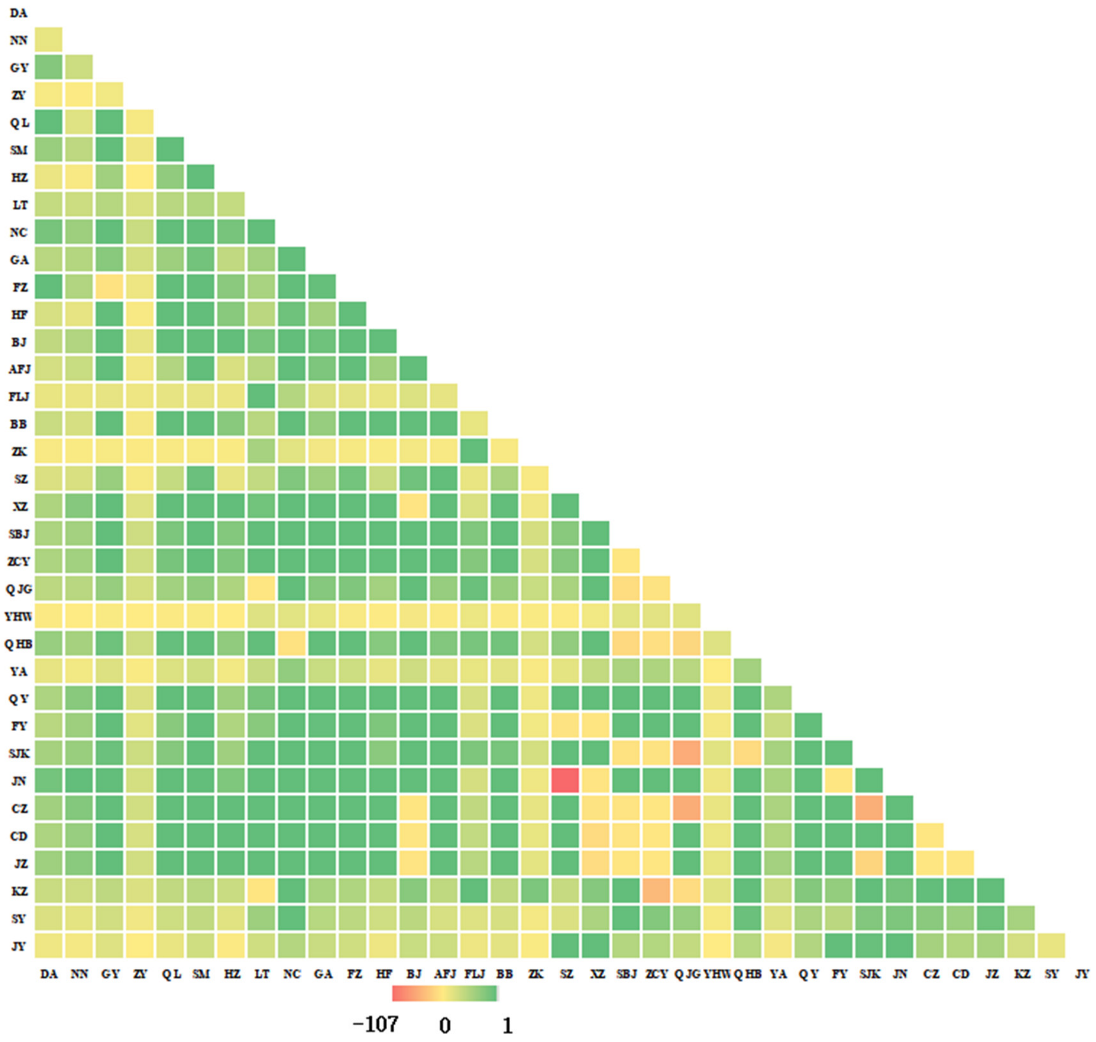

Figure S15 The Nm values among 35 populations of Cytb gene

3.3.5 Phylogenetic and Haplotype Network Analysis of Cytb Gene Sequence s

The maximum likelihood (ML) and Bayesian inference (BI) trees based on Cytb sequences showed consistent topologies, both dividing *R. pedestris* into t wo main clades (Figure S16, Figure S17). Clade 1 contained 18 haplotypes, pr imarily composed of populations from southwestern and southern China, includi ng all samples from Guiyang (GY) in Guizhou, Qujing (QL), Songming (SM), and Huize (HZ) in Yunnan, and Du'an (DA) and Nanning (NN) in Guangxi, a s well as partial samples from Liantang (LT) and Nanchang (NC) in Jiangxi, F uzhou (FZ) in Fujian, and Hefei (HF), Bobi (BJ), and Baoji (BB) in Anhui. T

he haplotype from Zunyi (ZY) in Guizhou was unique, clustering with northern populations but forming a distinct cluster. Clade 2 contained 56 haplotypes, encompassing all populations from central, eastern, northern, northwestern, and northeastern China, as well as some from southwestern and eastern China. The haplotype network (Figure S18) supported this pattern, incorporating 14 median vectors. Clade 1 included 18 haplotypes, with central haplotype H8 shared by 6 populations, likely the ancestral haplotype; H46, a unique haplotype from Nanning (NN) in Guangxi, was distant from the center, indicating genetic distinctiveness. Clade 2 included 56 haplotypes, with central haplotype H1 widely shared by 20 populations, serving as the ancestral haplotype. The nested star-like distribution of haplotypes in Clade 2 suggests rapid population expansion and diversification. The Zunyi (ZY) population formed a distinct cluster within Clade 2. Haplotypes from multiple populations in eastern China (e.g., Jiangxi, Fujian, Anhui) were distributed in both clades.

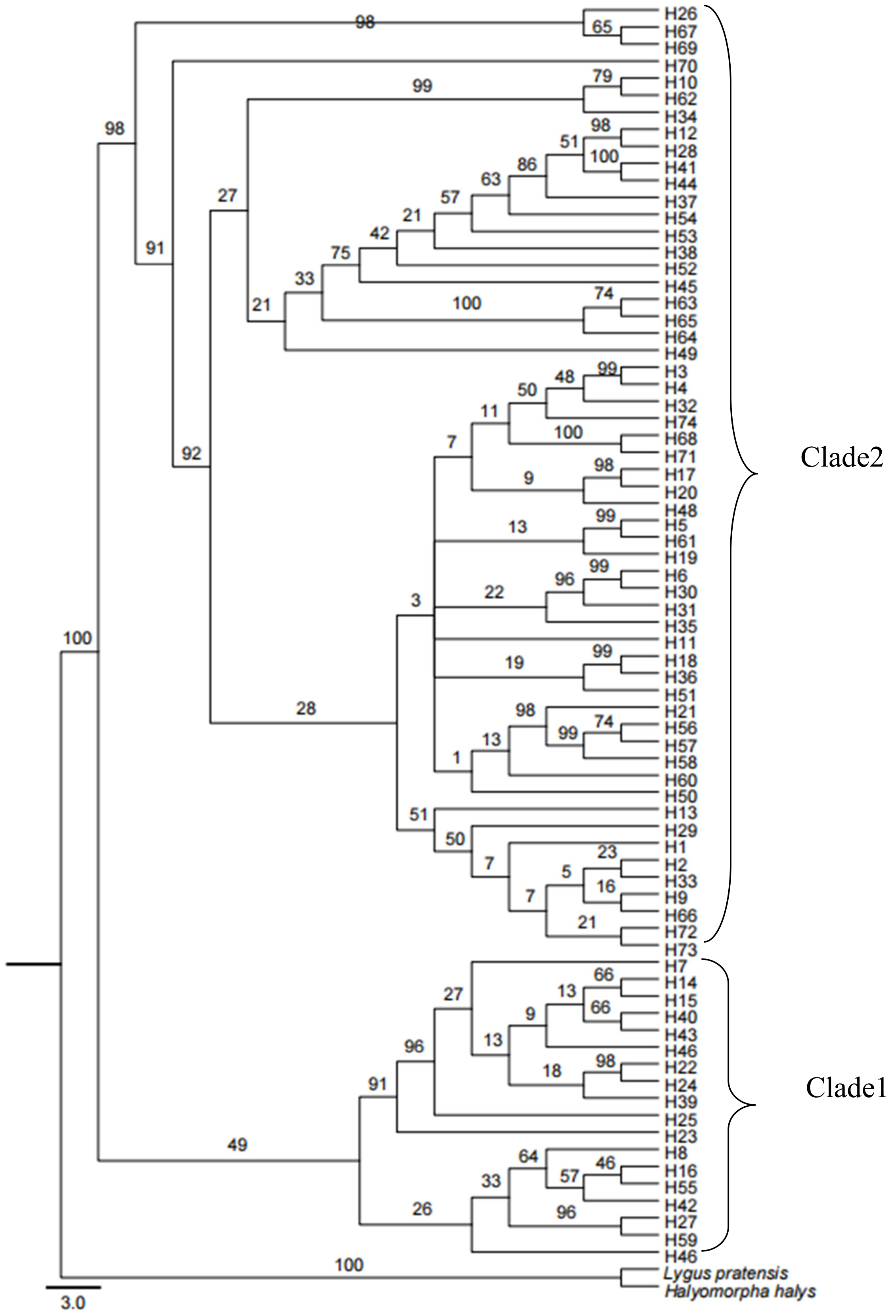

Figure S16 Cytb gene ML Phylogenetic trees based on 35 populations of *R. pedestris*

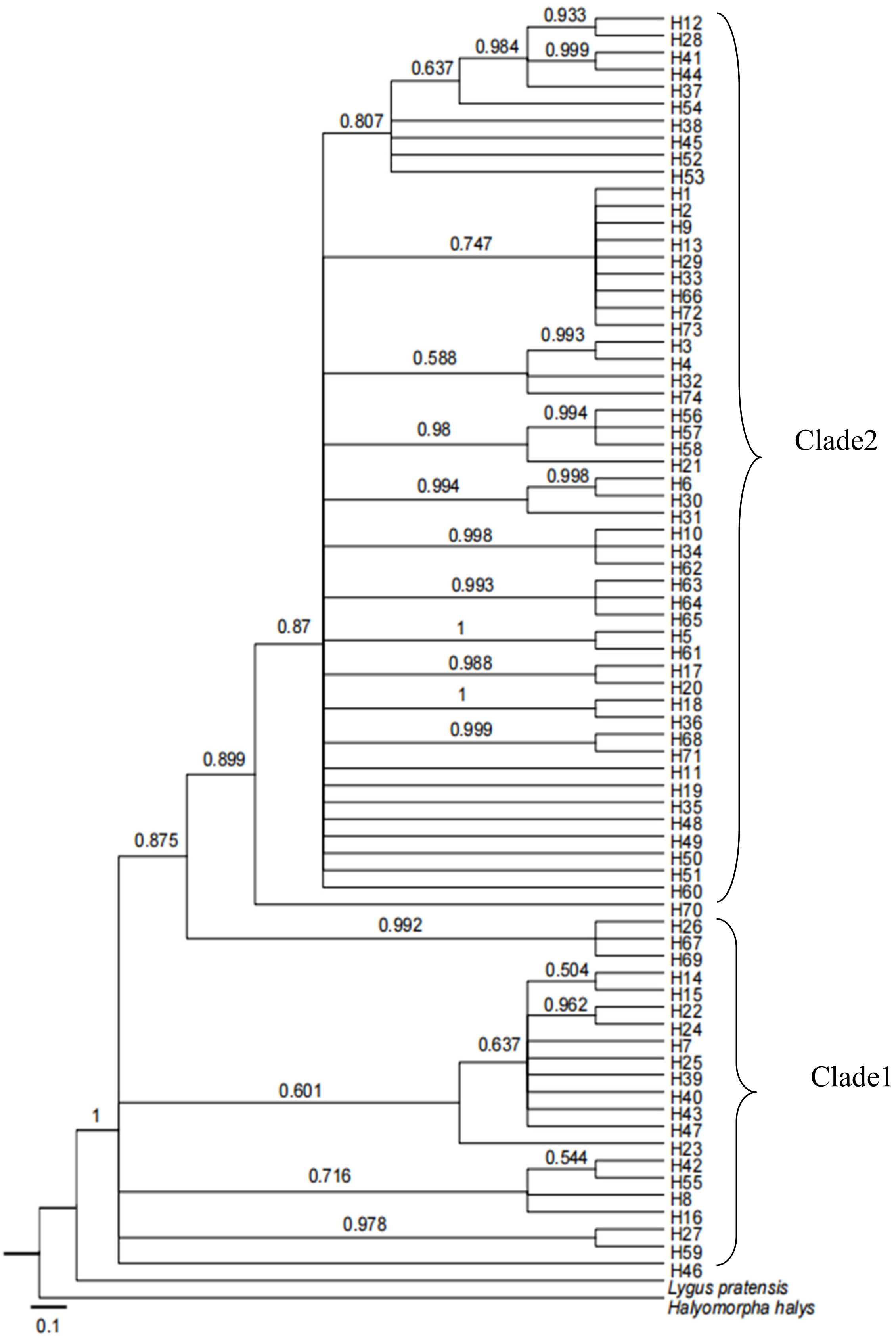

Figure S17 Cytb gene BI Phylogenetic trees based on 35 populations of *R. pedestris*

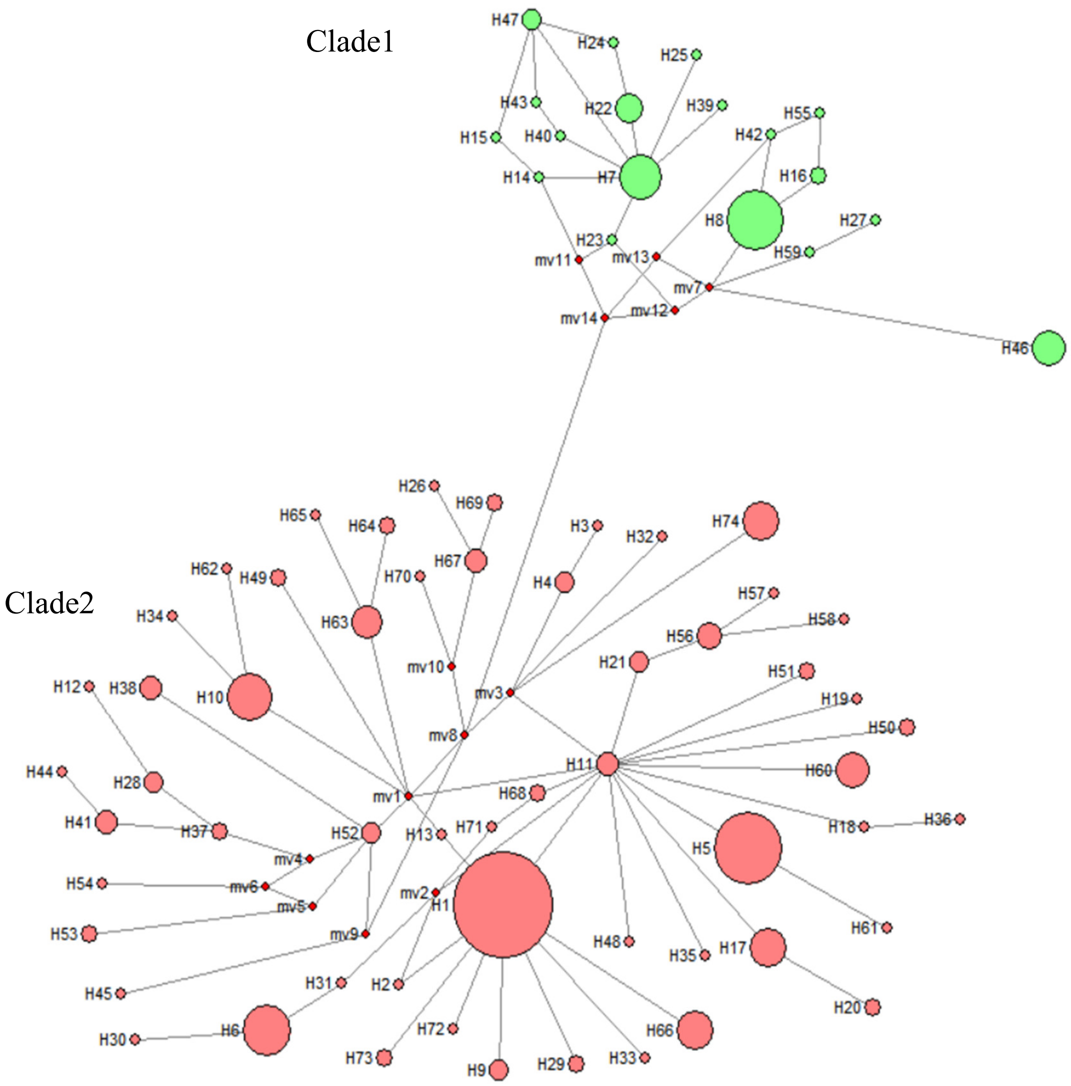

Figure S18 Haplotype network based on 35 populations of *R. pedestris*

3.4 Comparison of Analyses Across Mitochondrial Genes

Genetic diversity parameters were compared among COI, COII, Cytb, and the concatenated dataset (Table 4). All pairwise correlations were significant, with COI vs. Cytb, COI vs. concatenated, and Cytb vs. concatenated showing extremely high correlation ($P < 0.001$; $r = 0.997, 0.993, 0.999$, respectively). This indicates that results from single-gene analyses (especially COI or Cytb) are highly consistent with those from multi-gene concatenated sequences. Therefore, COI or Cytb alone can reliably reflect the genetic diversity and population structure of *R. pedestris* in phylogeographic studies.

Table S12 Comparison of genetic diversity parameters of three mitochondrial genes (COI,COII,Cytb) fragments and their combined sequences

**Indicators**	**COI**	**COII**	**Cytb**	**combined sequences**
COI	1	0.954*	0.997**	0.993**
COII		1	0.974**	0.977**
Cytb			1	0.999**
combined sequences				1

Note: ***P<0.001, **P<0.01, *P<0.05
